# The effects of polyunsaturated fatty acid (PUFA) administration on the microbiome-gut-brain axis in adolescents with anorexia nervosa (the MiGBAN study): study protocol for a longitudinal, double-blind, randomized, placebo-controlled trial

**DOI:** 10.1186/s13063-022-06413-7

**Published:** 2022-07-05

**Authors:** Lara Keller, Astrid Dempfle, Brigitte Dahmen, Samira Schreiber, Roger A. H. Adan, Nadia Andrea Andreani, Unna N. Danner, Albrecht Eisert, Sergueï Fetissov, Florian Ph. S. Fischmeister, Andreas Karwautz, Kerstin Konrad, Karlijn L. Kooij, Stefanie Trinh, Benny van der Vijgh, Annemarie A. van Elburg, Michael Zeiler, John Baines, Jochen Seitz, Beate Herpertz-Dahlmann

**Affiliations:** 1 Department of Child and Adolescent Psychiatry, Psychosomatics and Psychotherapy, University Hospital RWTH Aachen, Aachen, Germany; 2 Institute of Medical Informatics and Statistics, Kiel University, Kiel, Germany; 3 Brain Center Rudolf Magnus, University Medical Center Utrecht, Utrecht, Netherlands; 4 Max Planck Institute for Evolutionary Biology, Plön, Germany; 5 Altrecht Eating Disorders Rintveld, Altrecht Mental Health Institute, Zeist, The Netherlands; 6 Department of Clinical Psychology, Utrecht University, Utrecht, Netherlands; 7 Institute of Clinical Pharmacology, University Hospital RWTH Aachen, Aachen, Germany; 8 INSERM UMR-1239, University of Rouen Normandy, Rouen, France; 9 Institute of Psychology, University of Graz, Graz, Austria; 10 BioTechMed, Graz, Austria; 11 Department of Biomedical Imaging and Image-guided Therapy, Medical University of Vienna, Vienna, Austria; 12 Eating Disorders Unit at the Department of Child and Adolescent Psychiatry, Medical University of Vienna, Vienna, Austria; 13 Section for Neuropsychology, Department of Child and Adolescent Psychiatry, Psychosomatics and Psychotherapy, University Hospital RWTH Aachen, Aachen, Germany; 14 Institute of Neuroanatomy, University Hospital RWTH Aachen, Aachen, Germany; 15 Institute for Experimental Medicine, Kiel University, Kiel, Germany

**Keywords:** Anorexia nervosa, Gut microbiome, Gut microbiota, Microbiome-gut-brain axis, Supplementation, Polyunsaturated fatty acids, Clinical study, RCT, Inflammation, Gut permeability

## Abstract

**Background:**

Anorexia nervosa (AN) is a severe psychiatric disease that often takes a chronic course due to insufficient treatment options. Emerging evidence on the gut-brain axis offers the opportunity to find innovative treatments for patients with psychiatric disorders. The gut microbiome of patients with AN shows profound alterations that do not completely disappear after weight rehabilitation. In previous studies, the administration of polyunsaturated fatty acids (PUFA) resulted in effects that might be beneficial in the treatment of AN, affecting the microbiome, body weight and executive functions. Therefore, the MiGBAN study aims to examine the effects of a nutritional supplementation with PUFA on the gut microbiome and body mass index (BMI) in patients with AN.

**Methods:**

This is a longitudinal, double-blind, randomized, placebo-controlled trial. Within 2 years, 60 adolescent patients aged 12 to 19 years with AN will receive either PUFA or placebo for 6 months additional to treatment as usual. After 1 year, the long-term effect of PUFA on the gut microbiome and consecutively on BMI will be determined. Secondary outcomes include improvement of gastrointestinal symptoms, eating disorder psychopathology, and comorbidities. Additionally, the interaction of the gut microbiome with the brain (microbiome-gut-brain axis) will be studied by conducting MRI measurements to assess functional and morphological changes and neuropsychological assessments to describe cognitive functioning. Anti-inflammatory effects of PUFA in AN will be examined via serum inflammation and gut permeability markers. Our hypothesis is that PUFA administration will have positive effects on the gut microbiota and thus the treatment of AN by leading to a faster weight gain and a reduction of gastrointestinal problems and eating disorder psychopathology.

**Discussion:**

Due to previously heterogeneous results, a systematic and longitudinal investigation of the microbiome-gut-brain axis in AN is essential. The current trial aims to further analyse this promising research field to identify new, effective therapeutic tools that could help improve the treatment and quality of life of patients. If this trial is successful and PUFA supplementation contributes to beneficial microbiome changes and a better treatment outcome, their administration would be a readily applicable additional component of multimodal AN treatment.

**Trial registration:**

German Clinical Trials Register DRKS00017130. Registered on 12 November 2019.

**Supplementary Information:**

The online version contains supplementary material available at 10.1186/s13063-022-06413-7.

## Administrative information

Note: the numbers in curly brackets in this protocol refer to SPIRIT checklist item numbers. The order of the items has been modified to group similar items (see http://www.equator-network.org/reporting-guidelines/spirit-2013-statement-defining-standard-protocol-items-for-clinical-trials/).

**Table:** 

Title {1}	The effects of polyunsaturated fatty acid (PUFA) administration on the microbiome-gut-brain axis in adolescents with anorexia nervosa (the MiGBAN study): study protocol for a longitudinal, double-blind, randomized, placebo-controlled trial
Trial registration {2a and 2b}.	German Clinical Trials Register, DRKS00017130 (12 November 2019) https://www.drks.de/drks_web/navigate.do?navigationId=trial.HTML&TRIAL_ID=DRKS00017130
Protocol version {3}	11.4.2020 Version 1.5
Funding {4}	Federal Ministry for Education and Research (BMBF)/ EU programme ERA-NET NEURON
Author details {5a}	Lara Keller^1^, Astrid Dempfle^2^, Brigitte Dahmen^1^, Samira Schreiber^1^, Roger A.H. Adan^3^, Nadia Andrea Andreani^4^, Unna N. Danner^5,6^, Albrecht Eisert^7^, Sergueï Fetissov^8^, Florian Ph.S. Fischmeister^9,10,11^, Andreas Karwautz^12^, Kerstin Konrad^13^, Karlijn L. Kooij^3^, Stefanie Trinh^14^, Benny van der Vijgh^5^, Annemarie A. van Elburg^5,6^, Michael Zeiler^12^, John Baines^4,15^, Jochen Seitz^1*^, Beate Herpertz-Dahlmann^1*^ ^1^ Department of Child and Adolescent Psychiatry, Psychosomatics and Psychotherapy, University Hospital RWTH Aachen, Aachen, Germany; ^2^ Institute of Medical Informatics and Statistics, Kiel University, Kiel, Germany; ^3^ Brain Center Rudolf Magnus, University Medical Center Utrecht, Utrecht, Netherlands; ^4^ Max Planck Institute for Evolutionary Biology, Plön, Germany; ^5^ Altrecht Eating Disorders Rintveld, Altrecht Mental Health Institute, The Netherlands; ^6^ Department of Clinical Psychology, Utrecht University, Netherlands; ^7^ Institute of Clinical Pharmacology, University Hospital RWTH Aachen, Aachen, Germany; ^8^ INSERM UMR-1239, University of Rouen Normandy, France; ^9^ Institute of Psychology, University of Graz, Austria; ^10^ BioTechMed, Graz, Austria; ^11^ Department of Biomedical Imaging and Image-guided Therapy, Medical University of Vienna, Austria; ^12^ Eating Disorders Unit at the Department of Child and Adolescent Psychiatry, Medical University of Vienna, Austria; ^13^ Section for Neuropsychology, Department of Child and Adolescent Psychiatry, Psychosomatics and Psychotherapy, University Hospital RWTH Aachen, Aachen, Germany; ^14^ Institute of Neuroanatomy, University Hospital RWTH Aachen, Aachen, Germany; ^15^ Institute for Experimental Medicine, Kiel University, Kiel, Germany; * both authors contributed equally to this study
Name and contact information for the trial sponsor {5b}	Federal Ministry for Education and Research (BMBF) Heinemannstraße 2, 53175 Bonn, Germany
Role of sponsor {5c}	The funding of the BMBF had no role in the design of the study and will not have an impact on further stages of the trial, including collection, analysis and interpretation of data and the publication process.

## Introduction

### Background and rationale {6a}

Anorexia nervosa (AN) is a serious psychiatric disorder characterized by insufficient energy intake and malnourishment leading to severe underweight. Patients with AN show intense fear of gaining weight and often engage in activities that inhibit increase of body weight [1]. Adolescent girls and young women are particularly affected [2] and often spend a substantial portion of their teenage years fighting the disease [3]. The current state-of-the-art treatment is successful in less than 50% of all cases and many patients with AN suffer a chronic course of the disease with several relapses [4]. Moreover, AN has the highest mortality rate of all psychiatric diseases [5, 6]. Hence, there is a clear need for an improved treatment with novel therapeutic components to enhance patients’ quality of life and to achieve long-term recovery. The present trial aims to study the gut-brain axis as a promising new starting point to improve recovery rates in patients with AN.

In recent years, research on the gut-brain axis started investigating the interaction between the gut microbiome and the brain mediated via several pathways, including neural, endocrine, and immunological routes [7]. Multiple relationships between the gut microbiome and psychiatric diseases have been studied [8] showing for example profound perturbations in microbiome composition in patients with major depression [9, 10] or autism spectrum disorder [11, 12]. Furthermore, there is growing evidence that the gut microbiome plays an important role in AN [13]. Previous studies revealed a significant intestinal dysbiosis in AN, which was only partially alleviated with weight gain, e.g. lower abundances of Bacteroidetes and carbohydrate utilizing taxa as well as higher abundances of Firmicutes and Verrucobacteria [14–16]. The latter mucin degrading and protein-fermenting taxa are thought to increase intestinal wall permeability and potentially contribute to a “leaky gut” and thus trigger an immune response [17–19]. Transplantation of stool from patients with AN in germ-free mice led to reduced appetite and energy extraction in their offspring resulting in significantly reduced weight gain compared to the effects of transplantation of stool from healthy controls (HC). Furthermore, patient stool recipients showed increased anxiousness and compulsivity, further supporting the role of the microbiome-gut-brain axis in AN [20].

This emerging knowledge on gut-brain interaction provides a unique opportunity to develop innovative therapies for patients with psychiatric illnesses, in particular AN, since nutritional supplements like probiotics or omega-3 polyunsaturated fatty acids (PUFA) targeting the microbiome are promising candidates to indirectly influence these diseases via the microbiome-gut-brain axis [21, 22]. Previous trials have shown oral PUFA application to increase certain bacterial strains (e.g. Bacteroidetes) and decrease others (e.g. Firmicutes), which could potentially help ameliorate the dysbiosis observed in AN. Moreover, a reduction in gut permeability, inflammation parameters, depression, and anxiety is well documented for PUFA supplementation with low side effects for the recipient (see [23] for an overview). A first, small placebo-controlled study in patients with AN showed a tendency towards faster weight gain and better energy utilization following PUFA administration [24], but this needs to be tested systematically. In addition, Vesco and colleagues demonstrated that PUFA administration led to improvements in cognitive function in adolescents with mood disorders [25].

Based on these findings, the MiGBAN study intends to examine the effects of an intervention with omega-3 PUFA on the gut microbiome in patients with AN. Since AN is strongly (and more than any other psychiatric disease) connected with nutrition, malnourishment, and gastrointestinal symptoms, there is reason to believe that PUFA could help shift the microbiota composition, which in turn could reduce inflammation and increase body weight. Thus, supplementation with PUFA as an add-on for regular treatment could potentially result in positive effects on the course of the disorder and treatment success.

### Objectives {7}

The main objective of this trial is to longitudinally investigate the influence of 6 months PUFA administration on the gut microbiome composition of adolescent patients with AN over the course of hospital treatment and at 1 year follow-up compared to a placebo arm and in comparison to longitudinal changes in gut microbiome composition in healthy adolescents. Primary clinically relevant outcomes will be microbiome composition changes and weight gain. We furthermore want to study the effect of PUFA supplementation on eating disorder psychopathology, comorbidities, and gastrointestinal symptoms. To further deepen our understanding of the underlying pathophysiologic mechanisms, we want to elucidate the influence and interaction of the microbiota with the brain (microbiome-gut-brain axis) by conducting structural and functional Magnetic Resonance Imagining measurements as well as neuropsychological tests in our patients and in a group of HC. In addition, the interaction of the microbiome and inflammation markers, gut permeability, and hormones is investigated to study their role for the potential anti-inflammatory effect of PUFA in AN. Our hypotheses are that PUFA will have an anti-inflammatory effect mediated by the microbiota. Microbiome changes will furthermore lead to a reduction of the “leaky gut” and ameliorate energy utilization of the food consumed. Together, we expect these underlying mechanisms to lead to an increase in weight and a reduction in eating disorder and comorbid psychopathology as well as gastrointestinal symptoms, compared to the placebo group. Comparison to HC will further facilitate the understanding of the basic pathomechanisms of microbiome-gut-brain interaction in AN and help estimate the extent of normalization achieved by our intervention.

### Trial design {8}

**Fig. 1:**
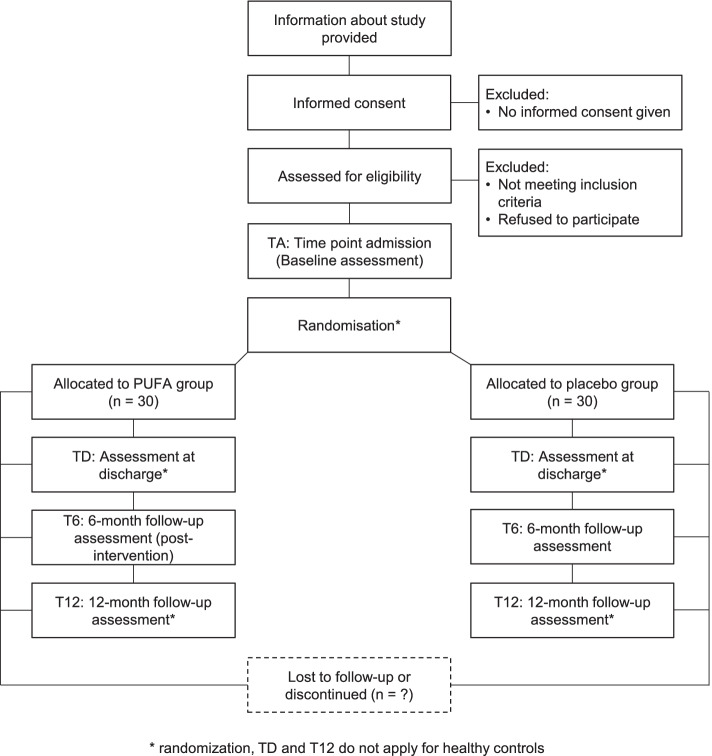
Overview of the planned trial design

The MiGBAN study is a longitudinal, double-blind, randomized, placebo-controlled superiority clinical trial with an observational HC group. Sixty AN patients will systematically be assessed directly after admission (measurement time point TA; pre-interventional baseline measurement), at discharge (TD), after 6 months (T6; post-intervention) and after 12 months (T12). Randomization to either PUFA or placebo will be performed as a stratified block randomization with a 1:1 allocation ratio. Stratification factors are sex, typical/atypical AN and MRI/non-MRI participation. For the 30 HC, only the measurement time points TA and T6 apply, and these participants will not receive any intervention (see Fig. 1 for an overview of the planned trial design). The trial was approved by the local Research Ethics Committee at the University Hospital RWTH Aachen (reference EK 194/19) and will be carried out in accordance with the Declaration of Helsinki. The study was registered at the German Clinical Trials Register (DRKS00017130, 12 November 2019).

## Methods: participants, interventions and outcomes

### Study setting {9}

The trial will be performed at the Department of Child and Adolescent Psychiatry, Psychosomatics and Psychotherapy, University Hospital RWTH Aachen (Germany). For the MRI measurements, a Siemens 3T Prisma scanner (Siemens AG, Erlangen, Germany) with a 64-channel head/neck coil located at the Department of Psychiatry, Psychotherapy and Psychosomatics, University Hospital RWTH Aachen will be used. The study is part of the international ERA-NET NEURON consortium “Microbiome Gut-Brain interaction in Anorexia Nervosa” (MiGBAN) with researchers from The Netherlands, Austria, France, and Germany. Another interventional study with a parallel study design will take place in Austria (Department of Child and Adolescent Psychiatry, Vienna General Hospital) with a supplementation of probiotics instead of PUFA [26].

### Eligibility criteria {10}

Inclusion and exclusion criteria are listed in Table 1.

**Table 1:** Inclusion and exclusion criteria

Inclusion criteria	Exclusion criteria (patient and HC group)
*- Patient group*: female and male patients with a diagnosis of typical or atypical AN (according to DSM-5) aged 12 to 19 years *- HC group*: adolescents aged 12 to 19 years with no current psychiatric disorder and no history of eating disorder	*-* Antibiotic use 6 weeks before inclusion *-* Diseases with an impact on the gastrointestinal tract (e.g. diabetes or inflammatory bowel disease) *-* Psychotic or bipolar disorder *-* Substance use disorder *-* Organic brain disease *-* IQ < 80 *-* Insufficient knowledge of German language *-* Pregnancy *- For the MRI measurements:* none of the standard MRI contraindications met (e.g. metal or electric implants in the body, claustrophobia)

### Who will take informed consent? {26a}

Trained research staff consisting of physicians and psychologists will obtain the written informed consent from both adolescents and their parents or caregivers. Afore, each person involved will receive detailed information about the study (including voluntariness of study participation) having the possibility to discuss questions. Participants and their parents or caregivers will need to give their written informed consent separately to participate in the MRI measurements after being thoroughly screened for any MRI contraindications and informed about the planned measurements and potential risks.

### Additional consent provisions for collection and use of participant data and biological specimens {26b}

On the consent form, participants will be informed that they have the right to withdraw their consent at any time. If consent is revoked, the participants’ data will be deleted or may be anonymized only and not deleted, if there still is a legal obligation to retain the data (e.g. after publication). Moreover, participants will be asked for permission for the research team to share relevant data with partners from the universities taking part in the research or from regulatory authorities, where relevant. This trial does involve collecting biological specimens for storage for third party use. No ancillary studies are planned in which participants’ data and biological specimens would be used. On the consent form, participants agree to be contacted again by trial investigators in case of further informational, consent-related purposes or future studies.

## Interventions

### Explanation for the choice of comparators {6b}

An administration of PUFA is hypothesized to positively influence the microbiome with beneficial effects on weight gain and psychopathology and could become an add-on for regular therapy in the long run to improve treatment of AN. However, it is ethically justifiable to administer a placebo to the patients of the non-intervention group (*N* = 30), since all patients participating in the trial will also receive treatment as usual (TAU) provided in our department.

The creation of a placebo intervention is crucial for successful blinding of participants and research staff. Therefore, the University Hospital Pharmacy was requested to produce a placebo capsule containing lactose and the same amount of vitamin D3 (15 μg) as the PUFA capsules. Just like the PUFA supplementation (see below) the placebo will be delivered twice a day with three capsules per dose. Vitamin D3 (90 μg/day) is administered to both groups to enable successful blinding as it is part of TAU for AN and also used as preservative in PUFA capsules.

### Intervention description {11a}

Thirty patients will receive daily oral doses of vegan over-the-counter PUFA (“Opti3” from Vegetology, Nottingham, UK) for a duration of 6 months. Similar to previous trials on depression, cognitive function, and weight gain [24, 25, 27, 28], the capsules contain both docosahexaenoic acid (DHA) and eicosapentaenoic acid (EPA) of vegan origin (algae). The patients will receive 6 capsules per day (containing in total 1500mg DHA, 900mg EPA, incl. 15μg vitamin D3) being administered in 3 capsules in the morning and 3 capsules in the evening. No serious adverse effects are known for PUFA and only low side effects like eructation, mild gastrointestinal symptoms, or a light taste of algae or fish in the mouth have been described [29]. In general, a supplementation with PUFA was found to be accepted and well-tolerated by patients with AN in previous studies [30–32].

### Criteria for discontinuing or modifying allocated interventions {11b}

Since only mild adverse effects are known for PUFA, it is expected that patients who are randomised to PUFA will directly benefit from this treatment. Theoretically, allergic reactions to one of the ingredients of both PUFA or placebo capsules could occur. In case of allergic reactions or other severe adverse events (SAEs), the intervention will be stopped immediately for an individual patient without any negative consequences. A physician will decide about the further procedure, yet a modification of the allocated intervention or dose is not intended. Any side effects or adverse events (AEs) will be regularly assessed and documented in standardized forms provided for this purpose. Participant request can end the intervention at any point, in which case participants would be asked to still voluntarily allow further data acquisition for an intention to treat analysis.

### Strategies to improve adherence to interventions {11c}

During inpatient treatment, the nursing staff will hand out the supplements to the patients ensuring a continuous administration. After discharge, a smartphone app will be recommended to remind patients of regularly taking the supplements and weekly medication boxes will ensure easy tracking of supplementation-intake when collecting boxes. Furthermore, parents will be asked to monitor the patients’ taking of the supplements after discharge and regular personal contacts to deliver the capsules and to ask for AEs will additionally contribute to the patients’ adherence and compliance to the intervention.

### Relevant concomitant care permitted or prohibited during the trial {11d}

In addition to the supplementation with either PUFA or placebo, all patients will receive TAU including nutritional, psychiatric and psychotherapeutic stepped care consisting of inpatient, day-patient and/or home treatment with weight rehabilitation at the Aachen Eating Disorder Unit. After discharge, outpatient treatment is highly encouraged. Antibiotics and other medication use during the trial will be closely monitored, pre- and probiotics use is explicitly discouraged, but any of these do not lead to termination of the trial intervention.

### Provisions for post-trial care {30}

As no harm from trial participation is expected, no ancillary or post-trial care actions are planned other than TAU. Study staff remains constantly available also after trial termination in case of questions or unexpected symptoms. Potential harm from trial participation is covered by in house and external insurance policies.

### Outcomes {12}

As co-primary outcomes, BMI (kg/m^2^) and overall microbiota community composition, will be analysed at 12-month follow-up. For microbiota community composition, the primary analysis will be based on beta-diversity within and between PUFA and placebo groups, and individual taxa abundance and alpha-diversity will also be considered. While microbiota characteristics do not have a direct translation into clinical relevance, weight restoration is one of the most important aims in the treatment of AN, justifying BMI as a co-primary outcome. Secondary measures include gastrointestinal symptoms, eating disorder psychopathology and comorbid symptoms (measured via questionnaires), physical activity (number of steps measured via fitness bracelets) as well as serum gut permeability and inflammatory markers. Finally, longitudinal changes of the above measures will be compared between patients with AN and HC using data from all four (two for HC) timepoints.

As previous research found clear alterations in cognitive functioning of patients with AN, especially learning abnormalities and aberrant reward processing (e.g. [33–35]), the trial incorporates neuropsychological tests on executive functioning, flexibility and probabilistic feedback learning all presented on a laptop using Inquisit 5 software (Millisecond Software, Seattle, USA, 2016). These neuropsychological measures are considered exploratory outcomes.

Since the study aims at contributing to a deeper understanding of the gut-brain axis in patients with AN, MRI measurements will be performed at three time points (TA, T6 and T12) in a subgroup of 30 patients and at two time points (TA and T6) for 20 HC. The MRI measurements will consist of structural (grey and white matter) scans and diffusion tensor imaging (DTI) and functional MRI scans. The functional MRI scans will include resting state measurements in order to study functional connectivity plus a probabilistic feedback learning task (based on [36, 37]) similar to the neuropsychological test.

### Participant timeline {13}

**Fig. 2:**
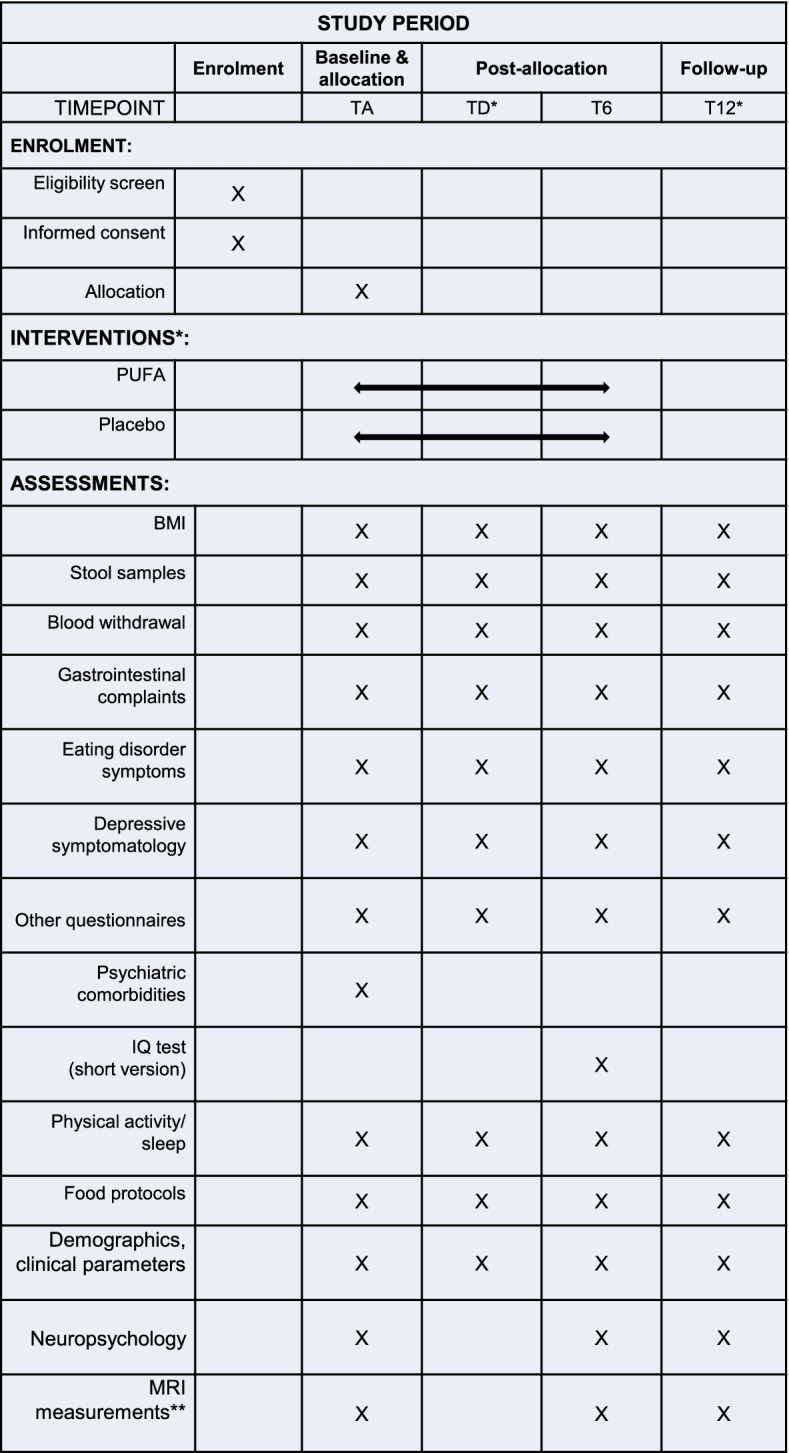
SPRIT figure: schedule of enrolment, interventions and assessments for patients and HC. Abbreviations: TA, time point admission; TD, time point discharge; T6, 6-month follow-up; T12, 12-month follow-up; BMI, body mass index; MRI, magnetic resonance imaging. *Intervention, TD and T12 do not apply for healthy controls. **For a subgroup of eligible participants only

See Fig. 2 for a schematic diagram of the time schedule for participants.

### Sample size {14}

So far, only one small study investigated the effects of providing PUFA compared to saturated fatty acids in patients with AN and found an effect size in weight gain of *d* = 1.0 [24]. Meta-analyses of probiotic interventions in depression and anxiety disorders revealed a pooled effect size of *d* = 0.68 for improvements in depressive symptoms and *d* = 0.66 for improvements in anxiety symptoms compared to placebo [38]. Thus, for the present study, we assumed an effect size of *d* = 0.8 for the effect of PUFA compared to placebo on BMI. To detect this effect using a two-sided *t*-test with a power of 80% at a significance level of *α* = 0.05, a sample size of 26 patients per group is needed. Considering a rate of loss-to-follow-up until 12 months of 10–15%, 30 patients per group, respectively 60 patients in total will be included in the trial. In case the final analysis will include 30 patients per group, the expected power (assuming an effect size of *d* = 0.8) will be 86%. For effect sizes of *d* = 1.0 and *d* = 0.65 as reported by previous studies mentioned above, the power will be 97% and 70%, respectively. No previous studies providing effect sizes for microbiome composition are available, but the direct effect of PUFA on microbiome composition can be expected to be at least as strong as the indirect effect on BMI.

### Recruitment {15}

Patient recruitment will take place in the Aachen Eating Disorder Unit. Every patient aged between 12 and 19 years admitted with a diagnosis of AN or atypical AN according to DSM-5 and his or her parents or caregivers will be provided detailed information about the study. Coupons for cinema visits and local stores are offered as remunerations for all as well as additional travel cost refunds for follow-up visits. The Aachen Eating Disorder Unit admits on average 50 patients with AN per year out of which 60 will be recruited in 2 years’ time (according to our previous study ANDI, in which two thirds of the eligible patients agreed to participate; [39]). Drop out until 12-month follow-up is estimated at no more than 10% according to previous clinical trials at our institution (6%, [39]; 4.5%, [40]). Age- and sex-matched HC will be recruited via advertisements and flyers in schools in Aachen and from participant lists of previous studies conducted at the Department of Child and Adolescent Psychiatry, Psychosomatics and Psychotherapy, University Hospital RWTH Aachen.

## Assignment of interventions: allocation

### Sequence generation {16a}

The allocation sequence that randomly assigns patients to either PUFA or placebo is based on computer-generated random numbers using the R package randomizeR [41]. A stratified block randomization with an allocation ratio of 1:1 is used including the following 5 strata: female typical AN/MRI, female typical AN/no MRI, female atypical AN/MRI, female atypical AN/no MRI, and male patients. The block size is 2 for female patients with a diagnosis of atypical AN and male patients and 4 for female patients with a diagnosis of typical AN.

### Concealment mechanism {16b}

Allocation concealment will be guaranteed since the administration of precalculated randomization lists is managed independently by the local Centre for Translational & Clinical Research Aachen (CTC-A) and research staff members will have no insight into the allocation sequence.

### Implementation {16c}

The allocation sequence is generated by Prof. A. Dempfle, Institute of Medical Informatics and Statistics, Kiel University, Kiel, Germany. She will deliver randomization lists (separate for each stratum) to the CTC-A where two staff members not involved in recruitment will be responsible for assigning patients to the intervention. These CTC-A staff members will prepare the respective intervention by filling a weekly medication box with PUFA supplements or placebo.

## Assignment of interventions: blinding

### Who will be blinded {17a}

All involved parties including patients, research staff members responsible for the inclusion and assessment of outcomes, as well as clinical staff delivering standard multimodal AN treatment will be blinded as supplement boxes arrive readily packed and labelled on the ward.

### Procedure for unblinding if needed {17b}

If SAEs or allergic reactions occur, the administration of PUFA or placebo will immediately be stopped until a physician will decide about the further procedure. Sealed envelopes containing the respective assignment are prepared for each patient and will be opened if the principal investigators consider it possible that knowledge of allocation might be beneficial for the patient’s safety.

## Data collection and management

### Plans for assessment and collection of outcomes {18a}

Body weight and height will be measured at each visit. Stool samples will be collected via stool-catching devices and immediately frozen at -80°C. Blood samples will be taken after an overnight fast. Self-reported gastrointestinal complaints are recorded with a shortened 17-item version of the Gastro-Questionnaire [42]. Changes in eating disorder symptoms are assessed by the Eating Disorder Inventory-2 (EDI-2; [43]) and the Eating Disorder Examination-Questionnaire (EDE-Q; [44]) and depressive symptomatology via the Beck Depression Inventory-II (BDI-II; [45]).

Other measures that could also act as confounding variables include obsessive-compulsive symptoms (Yale-Brown Obsessive Compulsive Scale, Y-BOCS; [46]), autistic traits (Social Responsiveness, SRS; [47]) and state anxiety (subscale “state” of the State-Trait Anxiety Inventory, STAI; [48]). In addition, sensitivity for reward and punishment measured by the Behavioral Inhibition System/ Behavioral Activation System scales (BIS/BAS scales; [49]) and impulsivity in form of a tendency to experience negative impulses or feelings in an exceedingly intense manner (subscale “urgency” of the UPPS-P Impulsive Behavior Scale; [50]) will be assessed. As further potential confounding variables comorbid psychiatric disorders will be identified at the first measurement time point using the Mini-International Neuropsychiatric Interview for Children and Adolescents (MINI Kid; [51]) by trained assessors. Six months after inclusion in the study, a short version of the Wechsler Intelligence Scale for children (WISC-V; [52]) and adults respectively for patients aged 17 years or older (WAIS-IV; [53]) will be conducted, i.e. only the two subtests “vocabulary” and “matrix reasoning” will be performed for an estimation of the total IQ.

Moreover, patients and HC will be asked to wear a fitness bracelet (model: Fitbit Flex or Fitbit Flex 2, Fitbit Inc., San Francisco, USA) for 48 h to investigate their physical activity (number of steps and active minutes) and sleep behaviour at all measurement time points.

To gain insight into the food consumption of patients and HC and to be able to perform correlation analyses between calorie content or nutrients and the microbiome composition, the amount of food eaten during 48 h prior to the respective measurement time point will be assessed by food protocols. Patients will additionally be asked to write down their food consumption during the last 48 h before admission to the inpatient treatment.

Furthermore, a series of other variables will be recorded in form of a case report form (CRF) including inter alia demographics and clinical parameters relating to the subtype, onset, and course of disease. For every participant all medication (especially antibiotics) or other supplements taken currently and before the respective measurement time point will be documented in detail.

In order to further examine aberrant cognitive functioning, the Iowa Gambling Task (IGT; [54]) and a Food Go/No-Go task [55] will be analysed. In addition, a probabilistic reversal learning (PRL) task (based on [56, 57]) will be performed to study feedback learning and reward processing in AN.

At TA, those patients participating in the MRI measurements will be asked to fill in a modified version [58] of the Edinburgh Handedness Questionnaire (EHQ; [59]).

The data collection will be conducted by thoroughly trained research staff members in cooperation with the CTC-A. Moreover, detailed standard operating procedures have been established to guarantee a consistent trial conduct over time irrespective of the person performing the data collection.

### Plans to promote participant retention and complete follow-up {18b}

The care system of our department usually accompanies patients with AN for a relatively long period of time after discharge including group psychotherapy offers and often individual psychotherapy, drug monitoring and weighing. Thus, most patients feel attached to our institution and regularly visit the clinic for appointments in the year after the actual discharge. This is advantageous to avoid drop-out in the trial by, e.g., scheduling follow-up meetings together with other appointments. If participants decide to discontinue the intervention, research staff members will endeavour to conduct the follow-up time points anyway including all outcomes to be able to perform intention-to-treat analyses if consent is not withdrawn. If participants refuse to participate in follow-up appointments in person, an effort will be made to inquire the most important outcomes (e.g. height and weight, eating disorder symptomatology, gastrointestinal complaints) via telephone and relevant questionnaires will be sent by mail. If necessary, stool samples can be sent to our department via express shipping as was successfully done in some cases in our pilot study [16].

### Data management {19}

Most of the data will be collected directly in digital form on a laptop, so that data entry and the associated errors are avoided. All other data collected using paper-pencil methods will be entered into a web-based database (Open Clinica LLC, MA, USA), while written CRFs will be stored separately enabling later comparison for quality assurance. For these data double entries followed by an automatic computer-based comparison of both versions will be performed and automatic range checks and forced entries (to prohibit missing values) will be conducted. For the neuroimaging data, an automated processing pipeline will be employed to perform timely quality control measurements and consistency checks so that a repetition in case of measurement artefacts will be possible.

### Confidentiality {27}

All data and samples obtained in the trial will be stored securely at the study site in a pseudonymized form allowing no conclusion to be drawn for single participants without the look-up table. Data and samples will be archived for at least 10 years after the end of the study and then anonymized. Imaging data will be anonymized via defacing, i.e. cutting off the front face section that could otherwise be used to reconstruct the participant’s face. The look-up table will be stored electronically, and access limited to staff members that handle inclusion and clinical data acquisition. All documents containing personal information (e.g. the informed consent form) will be stored separately from study records.

Before recruitment of participants started, a data protection impact assessment according to article 35 of the EU General Data Protection Regulation was made that evaluated the risks for rights and freedom of participants and staff in the present trial as low and acceptable.

### Plans for collection, laboratory evaluation and storage of biological specimens for genetic or molecular analysis in this trial/future use {33}

After collection, stool samples will be frozen, divided into three aliquots and stored at − 80°C. To analyse the microbiome composition frozen faecal samples collected from patients and HC will be subject to microbial genomic DNA extraction. Template DNA will be subject to 16S rRNA gene amplicon sequencing or directly (shotgun) sequenced using the Illumina MiSeq or NextSeq platforms, respectively. Blood serum samples will undergo centrifugation and will be separated into aliquots of 500μl and stored at − 80°C. Analysis for inflammatory markers, gut permeability markers and hormones will be conducted using ELISAs.

## Statistical methods

### Statistical methods for primary and secondary outcomes {20a}

#### Primary and secondary outcomes

For the co-primary outcome microbiome, the overall community composition will be compared between PUFA and placebo groups using non-parametric analysis based on beta-diversity (Permutational Multivariate Analysis of Variance Using Distance Matrices, [60]), including relevant baseline covariates age, sex, atypical/typical AN, and BMI at admission. For the other co-primary outcome measure BMI (at 12-month follow-up) a mixed-effects model repeated measures analysis will be used to test for superiority of the PUFA treatment vs. placebo. This model will include the covariates age, sex, atypical/typical AN, and BMI at admission. Any available further measurements of BMI (in particular at discharge and month six) will be used. As this is equivalent to a phase II trial, emphasis will be on the 95% confidence intervals of the effect size estimates. Moreover, *p* values will be calculated. For further exploratory analyses of microbiome data, see below.

Secondary outcomes such as eating disorder pathology will be analysed in an analogous way to the primary BMI analysis, additionally adjusting for baseline values of the outcome measure (at admission).

Furthermore, individual taxa abundances will be compared between PUFA and placebo groups using mixed models that account for, e.g. age, sex, medication and baseline or time-specific clinical covariates such as BMI and psychopathology. Correlations between diversity parameters and patient data within time points will be assessed via generalized linear models, whereas mixed models will be applied to assess relationships across time points. Exploratory analyses will associate microbiota community composition data at admission with clinical outcomes and identify characteristics of treatment responders vs. non-responders using “indicator-species” analysis and predictive machine learning algorithms (e.g. random forest models).

Finally, in addition to the randomized comparison between PUFA and placebo groups, a comparison between HC and patients (in both arms) will be done to assess both differences in gut microbiome composition and diversity between patients with AN and HC in the most acute phase of disease (at admission to inpatient treatment). Furthermore, the size of changes during and after treatment and weight rehabilitation in patients with AN compared to natural fluctuations of microbiome composition in healthy adolescents during normal development will be assessed. Again, mixed-effects models repeated measures analyses with an emphasis on descriptive reporting of confidence intervals of effect sizes will be performed.

#### Neuroimaging data

A Hierarchical Gaussian Filter (HGF) will be used to study reward sensitivity and cognitive flexibility during the functional MRI PRL task, in particular in the mesocorticolimbic circuitry, with a Bayesian brain perspective. The functional MRI analyses are planned to be conducted using SPM12 (Statistical Parametric Mapping, Wellcome Trust Centre for Neuroimaging, London, UK) and the HGF analyses will be performed with the help of open-source TAPAS software suite (developed by the Translational Neuromodeling Unit, Zurich, Switzerland) for MATLAB (The MathWorks Inc., MA, USA). The longitudinal study design allows to disentangle state and trait effects of AN that might be found in patients’ feedback learning and reward processing. Neuroplastic effects in resting state functional MRI connectivity will be analysed in the context of dynamic functional connectivity approaches with dynamic Bayesian network and graph analysis methods. Voxel-wise statistical analysis of white matter will be analysed concerning its global and regional fractional anisotropy by means of DTI analyses that will be carried out using TBSS (Tract-Based Spatial Statistics, [61]; part of FSL, [62]). Structural MRI will be analysed using Freesurfer image analysis suite (freely available for download online: http://surfer.nmr.mgh.harvard.edu/).

### Interim analyses {21b}

Not applicable as no interim analyses are planned in this trial.

### Methods for additional analyses (e.g. subgroup analyses) {20b}

Not applicable as no additional analyses are planned in this study.

### Methods in analysis to handle protocol non-adherence and any statistical methods to handle missing data {20c}

The primary analysis will be based on the intention-to-treat principle (ITT), including all randomized patients irrespective of the amount of treatment actually received. To follow ITT principle as closely as possible, all participants will be asked to participate in the end-of-treatment and follow-up assessment (at 6 months and 1 year), in particular regarding BMI measurement and providing a stool sample, even if they drop out of treatment, to minimize the amount of missing data. A per-protocol analysis will be performed as sensitivity analysis. To monitor the regular intake of PUFA intervention, the concentration of PUFA in the blood serum will be determined when drawing a blood sample at 6-month follow-up. In this clinical trial, any missing values of the primary outcome variables have to be considered to be missing not at random (MNAR). Patients with very poor response to the intervention and the standard multi-modal treatment of AN that is the same in both arms might be more likely to drop out of treatment and might be more likely not to provide data on the primary outcomes (loss to follow-up). Thus, imputation strategies or analysis methods that rely on the missing at random (MAR) assumption could be anti-conservative. However, every effort will be made to keep missing data as low as possible. In the primary analysis of the primary endpoint, we will use all available measures of the primary outcome BMI at all time points in a mixed-effects model repeated measures analysis.

Sensitivity analyses will be performed to investigate the potential impact of missing data, in particular, by performing a complete case analysis, a pre-specified conservative single imputation approach and by using multiple imputations.

### Plans to give access to the full protocol, participant-level data and statistical code {31c}

After termination of the study and publication of the results, access to the anonymized datasets will be provided upon reasonable request. Furthermore, the code and automated pipeline used for MRI analyses will be published on a public server.

## Oversight and monitoring

### Composition of the coordinating centre and trial steering committee {5d}

The trial steering committee consists of BHD and JS and will be responsible for the supervision of the study. LK acts as local coordinator of inclusion and data acquisition. A close cooperation with the local CTC-A has been established and specific tasks (e.g., blood sample management, participation in data and project management) are taken over by CTC-A employees. The Department of Child and Adolescent Psychiatry Aachen represented by BHD and JS will also function as coordinating centre for all sites of the ERA-NET NEURON consortium.

### Composition of the data monitoring committee, its role and reporting structure {21a}

The data monitoring committee (DMC) consists of AD and Nadine Krüger (CTC-A). The DMC is independent from the sponsor and the organizers.

### Adverse event reporting and harms {22}

Patients are asked to actively report and are interviewed weekly for any AEs that they might have perceived. Any AEs or SAEs irrespective of relationship to study intervention will be assessed and documented in standardized forms.

### Frequency and plans for auditing trial conduct {23}

Monthly meetings between psychologists and physicians responsible for conducting the study and a representative of the steering committee take place to supervise the practical implementation of the trial and to discuss potentially occurring problems. This procedure is independent from the sponsor, but not from investigators. In addition, a regular and close exchange of practical experience with the consortium partner in Austria will further contribute to a smooth flow of trial conduct.

### Plans for communicating important protocol amendments to relevant parties (e.g. trial participants, ethical committees) {25}

Any changes in the study protocol becoming necessary during the study will require approval by the local Research Ethics Committee at the University Hospital RWTH Aachen. Changes pertaining to already included participants will be communicated to them and their legal guardians.

### Dissemination plans {31a}

It is planned that results will be published in the form of articles in peer-reviewed scientific journals. There are no publication restrictions resulting from funding. After all results are published the anonymized trial data will be available to the public upon request. Moreover, a trial website will be created giving information about the project and the current status of results to the interested public.

## Discussion

Evidence on microbiome-gut-brain interaction is constantly growing indicating a connection between the gut microbiome and several psychiatric disorders [8, 63]. In AN the gut microbiome composition appears to be clearly altered which could potentially be associated with increased gut permeability and inflammation processes found in patients with AN. However, previous studies often lead to heterogeneous results [13, 18]. Thus, systematically investigating the role of an altered microbiome for the course and treatment of AN could lead to a deeper understanding of this disease. For example, it is unknown whether a longer duration of illness influences the composition of the microbiome and whether the dysbiosis itself becomes a self-perpetuating factor facilitating a transition to chronicity. Research conducted on microbiome-gut-brain interaction and its alteration, especially in the early stages of the illness, could be particularly important for the prognosis of this disorder. The gut microbiome could become a biomarker that might help identifying high-risk patients and taking appropriate steps at an early stage of treatment to provide the best treatment for each individual patient. Moreover, we aim to identify new effective therapeutic tools to improve the treatment and quality of life of patients suffering from this severe psychiatric disorder. AN even seems to be a quite “exemplary” disorder to study gut-brain interaction because there is no other mental disease in which nutrition and its changes play such a crucial role in addition to the microbiome-gut-brain axis. Therefore, during the treatment of AN, the gut microbiome might become an essential target during weight rehabilitation to influence food utilization, appetite, and gastrointestinal symptoms as well as neuropsychological functioning and behaviour. After supplementation with PUFA we expect alterations in the gut microbiome of patients approaching the composition of age-matched individuals without an eating disorder. In addition, we hope to improve gastrointestinal complaints and comorbid symptoms like mood and anxiety, to reduce inflammatory processes and to facilitate weight gain after treatment and at 12-month follow-up. All in all, PUFA supplementation could contribute to a better overall therapy outcome if the current clinical trial is successful, and its administration would be a readily applicable additional component of multimodal AN treatment.

## Trial status

Recruitment of patients began in January 2020 with protocol version 4.3.2. HC entered the trial at the end of June 2021 following postponing due to the COVID-19 pandemic. Recruitment of both patients and HC is still open and will approximately be completed end of June 2022.

## Supplementary Information

**Additional file 1.**

## Data Availability

The anonymized final datasets that will be generated in this study will be available from the corresponding author on reasonable request after the publication of the trial results.

## Abbreviations

AE: Adverse event
AN: Anorexia nervosa
BDI-II: Beck Depression Inventory-II
BIS/BAS: Behavioral Inhibition System/Behavioral Activation System
BMBF: Federal Ministry for Education and Research
BMI: Body mass index
COVID-19: Coronavirus disease 2019
CRF: Case report form
CTC-A: Center for Translational & Clinical Research Aachen
DHA: Docosahexaenoic acid
DMC: Data Monitoring Committee
DTI: Diffusion tensor imaging
DSM-5: Diagnostic and Statistical Manual of Mental Disorders
EDE-Q: Eating Disorder Examination-Questionnaire
EDI-2: Eating Disorder Inventory-2
EHQ: Edinburgh Handedness Questionnaire
EPA: Eicosapentaenoic acid
HC: Healthy controls
HGF: Hierarchical Gaussian Filter
IGT: Iowa Gambling Task
ITT: Intention-to-treat principle
MAR: Missing at random
MNAR: Missing not at random
MINI Kid: Mini-International Neuropsychiatric Interview for Children and Adolescents
MRI: Magnetic resonance imaging
PRL: Probabilistic reversal learning
PUFA: Polyunsaturated fatty acids
SAE: Serious adverse event
SPM12: Statistical Parametric Mapping
SRS: Social Responsiveness Scale
STAI: State-Trait Anxiety Inventory
TAU: Treatment as usual
TBSS: Tract-Based Spatial Statistics
UPPS-P: Urgency, Premeditation, Perseverance, Sensation Seeking, and Positive Urgency Impulsive Behavior Scale
WAIS-IV: Wechsler Adult Intelligence Scale - 4th Edition
WISC-V: Wechsler Intelligence Scale for Children - 5th Edition
Y-BOCS: Yale-Brown Obsessive-Compulsive Scale
