# Supplementary material for: The effects of polyunsaturated fatty acid (PUFA) administration on the microbiome-gut-brain axis in adolescents with anorexia nervosa (the MiGBAN study): study protocol for a longitudinal, double-blind, randomized, placebo-controlled trial
Source: Trials. 2022 Jul 5;23:545. doi: 10.1186/s13063-022-06413-7 (PMC9254435; doi:10.1186/s13063-022-06413-7)
Supplement: Supplementary file 1 — Additional file 1. [file 13063_2022_6413_MOESM1_ESM.zip › migban_consent_form_participants_parentsR1.pdf]

**Studienarzt**	**Studienzentrum**	**CTC-A-Nr.**
Prof. Dr. med. Beate Herpertz-Dahlmann	Klinik für Psychiatrie, Psychotherapie und Psychosomatik des Kindes- und Jugendalters, Uniklinik RWTH Aachen	18-118

Information zur Vorbereitung der mündlichen Aufklärung über die Teilnahme an einem Forschungsprojekt

*Untersuchung der Wirksamkeit der Verabreichung von mehrfach ungesättigten Fettsäuren auf das Mikrobiom bei Jugendlichen mit Anorexia nervosa vs. Placebo**

- MIGBAN -

Für die Eltern von Kontrollprobandin/proband: ______________________________

Liebe Eltern,

wir möchten Sie um Ihre Erlaubnis bitten, Ihre Tochter/Ihren Sohn fragen zu dürfen, an dem nachfolgend beschriebenen Forschungsprojekt teilzunehmen. Dieses Projekt wird durchgeführt, um weitere Erkenntnisse über die Erkrankung Anorexia nervosa (Magersucht) und deren Behandlungsmethode zu gewinnen. Das bedeutet, dieses Projekt wird zu Forschungszwecken durchgeführt.

Die Teilnahme an diesem Projekt ist freiwillig. Sie oder Ihre Tochter/Ihren Sohn können jederzeit ohne Angabe von Gründen Ihre Einwilligung widerrufen, ohne dass Ihnen oder Ihrer Tochter/Ihren Sohn dadurch Nachteile entstehen.

Im Folgenden möchten wir Sie über die Ziele und den Verlauf des Projektes informieren und erklären, warum Ihre und die Mitarbeit Ihrer Tochter/Ihres Sohns im Falle einer Teilnahme wichtig ist.

Wir bitten Sie, diese Information sorgfältig zu lesen und anschließend zu entscheiden, ob Ihre Tochter/Ihr Sohn mit Ihrem Einverständnis an diesem Projekt teilnehmen möchte oder nicht.

Wir möchten uns bereits im Vorfeld für Ihr Interesse bedanken.

1 Ziel des Forschungsprojektes

In unserem Projekt möchten wir untersuchen, ob die Einnahme eines Nahrungsergänzungsmittels, nämlich mehrfach ungesättigte Fettsäuren (Omega-3-Fettsäuren) einen Einfluss auf die Darmbakterien bei Jugendlichen mit Anorexia nervosa hat. Des Weiteren wollen wir den Stuhl oder Stuhlbestandteile Ihres Kindes in Tiermodelle transferieren, um dessen Auswirkungen zu erforschen. Die Ergebnisse dieses Forschungsprojektes sollen helfen, die Einflüsse der Ernährung auf die Darmbakterien zu verstehen und langfristig die Behandlung von Patienten und Patientinnen mit Anorexia nervosa zu verbessern.

Wir möchten insgesamt 60 stationäre Patientinnen/Patienten mit Anorexia nervosa und 30 gesunde Mädchen/Jungen im Alter zwischen 12 und 20 Jahren für das Projekt untersuchen. Von den 60 Patientinnen/Patienten erhalten 30 Patientinnen/Patienten das Nahrungsergänzungsmittel und die anderen 30 Patientinnen/Patienten ein Placebo. Placebo bedeutet ein Scheinmittel, das keinerlei Wirkstoff enthält**.** Als Kontrolle suchen wir gesunde Kontrollprobanden, die kein Nahrungsergänzungsmittel nehmen sollen.

2 Was sind Omega-3-Fettsäuren?

Omega-3-Fettsäuren gehören zu den mehrfach ungesättigten Fettsäuren und sind für den Körper lebensnotwendig. Der Körper kann Omega-3-Fettsäuren nicht selbst produzieren und muss sie daher über die Nahrung aufnehmen. Sie kommen z. B. in pflanzlichen Lebensmitteln wie Lein, Raps und Walnuss vor und stecken vor allem in Algen und fetten Meeresfischen. Wir verwenden frei verkäufliche, ausschließlich vegane Omega-3 Fettsäuren aus Algen (Opti3 Omega-3 EPA & DHA von Vegetology, Nottingham, UK). Man geht heute davon aus, dass Omega-3-Fettsäuren auch eine positive Wirkung auf das Gehirn haben.

3 Ein- und Ausschlusskriterien

Teilnehmen können gesunde Jugendliche ohne aktuelle psychiatrische Erkrankungen und ohne Essstörungsvergangenheit zwischen 12 und 20 Jahren mit Einverständnis der Eltern oder des gesetzlichen Vormundes bei Minderjährigen ohne sorgeberechtigte Eltern. Nicht teilnehmen können Jugendliche mit organischen Hirnerkrankungen, IQ <80, unzureichenden Deutschkenntnissen, organischen Erkrankungen mit Einfluss auf den Magen-Darmtrakt, wie z.B. Diabetes oder entzündlichen Darmerkrankungen, Schwangerschaft, sowie systemischer Antibiotikaeinnahme in den letzten 6 Wochen.

4 Was kommt während der Teilnahme an dem Projekt auf Ihre Tochter/Ihren Sohn zu?

Zunächst überprüft der zuständige Arzt, ob Ihre Tochter/Ihr Sohn die Voraussetzungen zur Teilnahme an dem Projekt erfüllt. Nach ausführlicher mündlicher und schriftlicher Aufklärung zum Forschungsvorhaben durch den Studienarzt und Erhalt einer von Ihnen und Ihrer Tochter/Sohn unterschriebenen Einwilligungserklärung wird Ihre Tochter/Ihr Sohn in das Projekt eingeschlossen.

Anschließend werden zunächst folgende Daten von Ihrer Tochter/ Ihrem Sohn erhoben/dokumentiert: Alter, Größe, Gewicht, aktuelle Medikation und anderweitige Erkrankungen.

Wir möchten Ihre Tochter/Ihren Sohn an drei Zeitpunkten des Forschungsprojektes untersuchen. Der erste Untersuchungszeitpunkt findet nach Einschluss in das Projekt statt, der zweite nach 6 und der letzte nach 12 Monate, nachdem Ihre Tochter/Ihr Sohn mit der Studie begonnen hat.

Als gesunder Kontrollproband wird Ihrer Tochter/Ihrem Sohn kein Prüfpräparat verabreicht.

Die geplanten Untersuchungen werden in den nachfolgenden Punkten genauer erläutert.

4.1 Fragebögen & neuropsychologische Tests

Bei jedem Untersuchungszeitpunkt bekommt Ihre Tochter/Ihr Sohn kurze Fragebögen zu ihrem/seinem Wohlbefinden und Symptomen (Essstörungen, Angst, Depression) ausgehändigt bzw. füllt diese an einem Computer aus (ca. 30 min). Außerdem erfolgen neuropsychologische Tests am Computer (ca. 40 min). Insgesamt handelt es sich um 8 – 9 Fragebögen pro Untersuchungszeitpunkt. Abhängig davon, ob Ihr Kind an der fMRT-Untersuchung teilnimmt. Des Weiteren werden Sie gebeten, beim Studienstart und ein Jahr später einen Fragebogen zur Erfassung sozialer Reaktivität (SRS) auszufüllen. Dieser Test dient dazu die Wechselseitigkeit in sozialen Interaktionen bei Ihrem Kind festzustellen. Ein Mangel kann ein erstes Anzeichen für soziale kognitiven Funktionen sein und ist im Rahmen von der Autismus-Diagnostik relevant.

4.2 Interview zu klinischen Symptomen

Dieses Interview möchten wir bei Aufnahme in die Studie mit Ihrer Tochter/Ihrem Sohn führen, es dauert ca. 30 min.

4.3 Stuhlproben

Weiterhin möchten wir Ihre Tochter/Ihren Sohn bitten, zu jedem Untersuchungszeitpunkt eine Stuhlprobe abzugeben, um die Darmbakterien zu untersuchen. Dabei wird der Stuhl von Ihrer

Tochter/Ihrem Sohn in einer Plastik-Auffangvorrichtung (Fecotainer) gesammelt, die unter den Toilettensitz eingelegt werden kann und luftdicht verschlossen wird.

4.4 Blutproben

An drei Zeitpunkten benötigen wir Blutproben von Ihrer Tochter/Ihrem Sohn. Im Blut werden Entzündungszeichen, Hormone, Botenstoffe, Fettsäuren und Blutzellen untersucht. Eine Untersuchung der Gene Ihres Kindes findet nicht statt. Es sind keine für Ihr Kind individuell relevante, krankheitsbezogene Befunde zu erwarten, die Untersuchungen dienen einzig der Forschung. Die Blut- und Stuhl-Proben werden in Tiefkühltruhen der KJP anonymisiert gelagert und für die Analysen ebenfalls anonym an unsere Kooperationspartner verschickt. Sie werden nach Abschluss der Analysen, spätestens aber nach 10 Jahren vernichtet.

4.5 Ernährungstagebuch

Ihre Tochter/Ihr Sohn wird gebeten, zwei Tage vor jeder Kontrolle ein Tagebuch über ihre/seine verzehrten Nahrungsmittel zu führen und dieses mitzubringen. Sollte sie/er innerhalb von 6 Wochen vor diesen Untersuchungen Medikamente oder in den letzten 6 Monaten ein Antibiotikum oder Medikamente eingenommen haben, möchten wir Sie bitten, auch dies aufzuschreiben.

4.6 Bewegungsmessung (Aktimetrie)

An allen Zeitpunkten wird mittels eines Armbandes ("Aktimeter") für drei Tage die Bewegung gemessen. **Funktionelles und strukturelles MRT (Magnetresonanztomographie)**

Bei den Probandinnen/Probanden ohne Gegenanzeigen wie z.B. eine große Metallzahnspange (siehe auch separate Fragebögen) möchten wir bei Aufnahme sowie 6 und 12 Monate nach Aufnahme eine MRT (Magnetresonanztomografie oder Kernspin)-Untersuchung des Kopfes im Forschungsscanner der Klinik für Psychiatrie (Siemens, 3 Tesla) im Uniklinikum Aachen durchführen.

Bei der MRT-Untersuchung wird Ihre Tochter/Ihr Sohn auf dem Rücken liegend in den Gerätetunnel, der sowohl am Kopf- als auch am Fußende offen ist, hineingefahren. Der Gerätetunnel besteht aus einem starken Magneten, der in Verbindung mit elektrischen Spulen innere Bilder des Körpers erzeugt. Die Untersuchung ist nach heutigem Wissen ungefährlich, lediglich sehr laut, sodass Ihre Tochter/Ihr Sohn Ohrenstopfen bekommt. Eine Strahlenbelastung ist mit dieser Untersuchung nicht verbunden. Die gesamte Untersuchungszeit im MRT beträgt ca. 60 Minuten. Diese beinhaltet anatomische Messungen (T1, T2 und diffusions-gewichtete Scans) sowie eine „Resting State" Messung, also eine Messung der Gehirnfunktion in Ruhe. Bei allen diesen Messungen soll ihre Tochter/Ihr Sohn lediglich entspannt und ruhig daliegen und sich möglichst wenig bewegen. Bei zwei weiteren funktionellen Messungen wird sie/er gebeten, wiederholt eine von zwei Figuren auszuwählen. Für die „richtige" Wahl können Punkte gewonnen werden; welche Figur die „Richtige" ist, wechselt nach einem bestimmten Muster, das die Teilnehmer herausfinden sollen. Zwischen

den Messungen erfolgt immer ein kurzer Kontakt mit den Teilnehmern, ob alles in Ordnung ist, und er/sie wird über die nächste Messung informiert. Während der einzelnen Messungen kann jederzeit mittels eines „Rufballs“ mit dem Untersuchungsleiter Kontakt aufgenommen werden.

Wir machen Sie und Ihre Tochter/Ihren Sohn darauf aufmerksam, dass bei ca. 3% aller jüngeren gesunden Menschen Normvarianten der Anatomie oder Strukturabweichungen bestehen, die in der Regel ohne Krankheitswert sind. Bei älteren Menschen ist diese Zahl höher. In seltenen Fällen können aber auch einmal behandlungsbedürftige Zufallsbefunde entdeckt werden.
Qualität und Anzahl der bei Ihrer Tochter/Ihrem Sohn dann erstellten Schnittbilder sind allerdings nicht geeignet, Strukturabweichungen sicher zu erfassen oder genauere Aussagen über die Art solcher Veränderungen zu machen, weil die eingestellten Betriebswerte auf das spezielle Forschungsziel, z. B. das funktionelle Verhalten einzelner Forschungsabschnitte, ausgerichtet sind. Sollte sich trotzdem ein Hinweis auf einen Befund mit möglichem Krankheitswert ergeben, müssen Sie sich damit einverstanden erklären, dass Ihnen dies vom Untersuchungsleiter mitgeteilt wird. Der Studienleiter leitet dann zur raschen Abklärung in Abstimmung mit dem(n) Sorgeberechtigten weiterführende Untersuchungen in die Wege, die mindestens aus einer neuropädiatrischen Untersuchung und einer dann diagnostischen MRT bestehen, die durch einen Facharzt für Neuroradiologie beurteilt werden wird. Sollte sich ein behandlungsbedürftiger Befund ergeben, kann das für Ihre Tochter/Ihr Sohn – dann als Patient/in – soziale und psychische Folgen haben. Im Bereich der Forschung wird aber zurzeit weniger als 1 Fall von 100 beobachtet, bei dem weitere Untersuchungen empfohlen werden.

5 Welche Risiken gibt es?

Bei Einhaltung aller Sicherheitsvorschriften und Ausschlusskriterien gilt die Magnetresonanz-Tomographie zum heutigen Kenntnisstand als weitgehend ungefährlich. Bei einer Blutentnahme besteht grundsätzlich das Risiko einer Infektion der Einstichstelle, Nervenverletzungen, Hämatome, Schwindel und Unwohlsein

6 Mögliche Bedeutung der Projektergebnisse und Risiko/Nutzenabwägung

Wie bei allen Experimenten, sind die Ergebnisse zu Beginn eines Forschungsvorhabens nicht absehbar. Die Teilnahme an dem Projekt hat daher voraussichtlich keinen unmittelbaren persönlichen Nutzen für Ihre Tochter/Ihren Sohn. Allerdings könnte unser Projekt für zukünftige Patienten und Patientinnen wichtige Erkenntnisse und eine wesentliche Verbesserung der Behandlung dieser schweren Krankheit erbringen. Mit der Teilnahme an dem Projekt könnte Ihre Tochter/Ihr Sohn vielen zukünftigen Patienten und Patientinnen helfen.

7 Aufwandentschädigung

Für jeden Untersuchungszeitpunkt erhält Ihre Tochter/Ihr Sohn Bargeld in Höhe von 30 EUR plus Fahrtkosten.

8 Personenbezogene Informationen und Ergebnisse

Die für das Forschungsprojekt wichtigen Daten werden in pseudonymisierter Form gespeichert, ausgewertet und innerhalb der Forschergruppe weitergegeben. Pseudonymisiert bedeutet, dass keine Angaben von dem Namen Ihrer Tochter/Ihrem Sohn oder ihre/seine Initialen verwendet werden, sondern nur ein Nummern- und/oder Buchstabencode. Die Namensliste, die allein eine Zuordnung der Daten bzw. der Ergebnisse zu ihrer/seiner Person gestattet, verbleibt unter Verschluss in unserer Klinik. Die Liste wird nach der von der Deutschen Forschungsgemeinschaft geforderten und gesetzlich vorgeschriebenen zehnjährigen Nachweispflicht gelöscht. Bis zu diesem Zeitpunkt wird die Namensliste in einem verschlossenen Raum in unserer Klinik aufbewahrt.
Die im Rahmen des Projektes erhobenen Daten und medizinischen Befunde von Ihrer Tochter/Ihrem Sohn werden von einem elektronischen Datensystem erfasst und statistisch ausgewertet. Nach Beendigung des Projektes werden alle Daten nach den derzeit gültigen Richtlinien entsprechend gespeichert und archiviert. Die Verantwortung für die Einhaltung des Datenschutzes im Rahmen des elektronischen Datensystems liegt bei dem Institut für Medizinische Informatik am Universitätsklinikum Aachen, Pauwelsstr. 30, 52074 Aachen. Die Bearbeitung der erhobenen Daten erfolgt in Verantwortung von Prof. Dr. med. Beate Herpertz-Dahlmann/PD Dr. med. Jochen Seitz, Klinik für Psychiatrie, Psychotherapie und Psychosomatik des Kindes- und Jugendalters der Uniklinik RWTH Aachen, Neuenhofer Weg 21, 52074 Aachen. Sie haben das Recht, Einsicht in die Daten zu nehmen, die während der Studie erhoben werden. Sollten Sie dabei Fehler in den Daten feststellen, haben Sie das Recht, diese durch den Studienarzt korrigieren zu lassen.
Weiterhin haben Sie das Recht auf Auskunft und Überlassung einer Kopie der Daten. Sie haben darüber hinaus das Recht, sich bei einer Aufsichtsbehörde (siehe Punkt 14, Adressen und Kontakte) über den Umgang mit den Daten Ihrer Tochter/Ihres Sohns zu beschweren.
Sie und Ihre Tochter/Ihr Sohn treffen Ihre Entscheidung nach der mündlichen Aufklärung freiwillig und können Ihr Einverständnis jederzeit zurücknehmen, ohne dass Ihrer Tochter/Ihrem Sohn daraus Nachteile entstehen.

Bei wissenschaftlichen Forschungsprojekten werden persönliche Daten und medizinische Befunde über Ihre Tochter/Ihren Sohn erhoben. Dabei ist gesetzlich festgelegt, dass diese studienbezogenen Daten nur ohne Namensnennung gespeichert, weitergegeben und ausgewertet werden dürfen, und zwar:

1.) an den Auftraggeber der Studie zur wissenschaftlichen Auswertung;

Anschrift der Auftraggeber:

Prof. Dr. med. Beate Herpertz-Dahlmann/PD Dr. med. Jochen Seitz,
Klinik für Psychiatrie, Psychotherapie und Psychosomatik des Kindes- und Jugendalters,
Uniklinik RWTH Aachen,
Neuenhofer Weg 21, 52074 Aachen
Tel. 0241/8089171

2.) Außerdem kann ein autorisierter und zur Verschwiegenheit verpflichteter Beauftragter des Auftraggebers in die beim Studienarzt vorhandenen personenbezogenen Daten Einsicht nehmen, soweit dies für die Überprüfung der Studie notwendig ist.

9 Versicherung

Die Uniklinik RWTH Aachen und deren an der Studie mitwirkende Mitarbeiter sind haftpflichtversichert für den Fall, dass Ihre Tochter/Ihr Sohn durch deren Verschulden einen Schaden erleiden. Da Ihre Tochter/Ihr Sohn für die zwei Nachuntersuchungen nochmal in die Klinik eingeladen wird, gibt es zusätzlich für alle Teilnehmer eine Reise-Unfallversicherung für den Weg zur Klinik und zurück mit den Höchstversicherungssummen 100.000 EUR bei Invalidität und 50.000 EUR bei Tod.

10 Finanzierung der Studie

Die Studie wird vom Bundesministerium für Bildung und Forschung finanziert. Sie ist Teil eines europäischen Forschungsprojektes.

11 Freiwilligkeit/Nichtteilnahme

Wenn Sie Ihre Tochter/Ihr Sohn nicht an dem Projekt teilnehmen lassen möchten, entstehen Ihnen und Ihrer Tochter/Ihrem Sohn keinerlei Nachteile. Ihre Entscheidung zur Teilnahme bzw. der Rückzug Ihrer Einwilligung zur Teilnahme Ihrer Tochter/Ihres Sohns an diesem Projekt hat keinen Einfluss auf eine etwaige reguläre medizinische Versorgung Ihrer Tochter/Ihres Sohns. Ihre/Seine Teilnahme ist zu jeder Zeit freiwillig.

12 Haben Sie weitere Fragen?

Sollten Sie noch weitere Fragen zum Projektverlauf, zu Ihren Rechten oder zum Versicherungsschutz haben, wenden Sie sich bitte an einen der Studienärzte.

13 Information über neue Erkenntnisse

Ihr Studienarzt wird Sie und Ihre Tochter/Ihr Sohn in einer angemessenen Frist auch über jede weitere wichtige, während des Projektes bekanntwerdende Information in Kenntnis setzen, die Ihre Einwilligung zur weiteren Teilnahme beeinflussen könnte.

Etwaige relevante Befunde werden Ihnen und Ihrer Tochter/Ihrem Sohn im Rahmen der medizinischen Betreuung unmittelbar zur Kenntnis gebracht. Die spätere Auswertung des gesamten Projektes wird Ihnen und Ihrer Tochter/Ihrem Sohn auf Wunsch mitgeteilt.

Die Ergebnisse dieses Projektes werden nach der Analyse in einem wissenschaftlichen Artikel verarbeitet und in einer medizinischen Fachzeitschrift veröffentlicht. Hierüber werden Sie nicht persönlich informiert. Die Daten Ihrer Tochter/Ihres Sohns werden dabei zu keiner Zeit namentlich veröffentlicht bzw. an Dritte weitergegeben.

14 Adressen und Kontakte

Studienzentrum:

Prof. Dr. med. Beate Herpertz-Dahlmann/PD Dr. med. Jochen Seitz
Klinik für Psychiatrie, Psychosomatik und
Psychotherapie des Kindes- und Jugendalters
Uniklinik RWTH Aachen
Neuenhofer Weg 21
52074 Aachen
Tel: 0241 80 89171
Fax: 0241 80 82544

Datenschutzbeauftragter der Projektleitung und des Studienzentrums:
Joachim Willems
Uniklinik RWTH Aachen
Pauwelsstraße 30
52074 Aachen
Tel: 0241 80 89051
Fax: 0241 80 3389051

Datenschutzaufsichtsbehörde (Projektleitung und Studienzentrum)
Landesbeauftragte für Datenschutz und Informationsfreiheit (LDI)
Nordrhein-Westfalen
Postfach 20 04 44
40102 Düsseldorf
Tel: 0211/38424-0
Fax: 0211/38424-10

15 Einwilligungserklärung

Probanden Nr.:

Hiermit willige ich freiwillig in die beschriebene Teilnahme an der Studie und der Erhebung und Verarbeitung der personenbezogenen Daten meiner Tochter/meines Sohns ein. Ich bin ausreichend informiert worden und hatte die Möglichkeit, Fragen zu stellen. Über die Folgen eines Widerrufs der Gesamtteilnahme oder der datenschutzrechtlichen Einwilligung bin ich aufgeklärt worden. Eine Kopie der schriftlichen Aufklärung und Einwilligung sowie der Versicherungsbestätigung und –bedingungen der Wege-Unfall-Versicherung habe ich erhalten.

15.1 Datenschutz

Bei wissenschaftlichen Projekten werden persönliche Daten und medizinische Befunde über Sie/Ihre Tochter/Ihren Sohn erhoben. Die Weitergabe, Speicherung und Auswertung dieser projektbezogenen Daten erfolgt nach gesetzlichen Bestimmungen und setzt vor Teilnahme an dem Projekt die folgende freiwillige Einwilligung voraus:

1. ☐*(bitte ankreuzen)* **Ich erkläre mich damit einverstanden, dass im Rahmen dieses Projektes personenbezogene Daten, insbesondere Angaben über die Gesundheit und ethnische Herkunft, über mich/meine Tochter/meinem Sohn erhoben und in Papierform sowie auf elektronischen Datenträgern in der Klinik für Psychiatrie, Psychosomatik und Psychotherapie des Kindes- und Jugendalters, Uniklinik RWTH Aachen aufgezeichnet werden.**

Soweit erforderlich, dürfen die erhobenen Daten pseudonymisiert (d.h. die Daten können ohne Hinzuziehung zusätzlicher Informationen nicht mehr einer spezifischen betroffenen Person zugeordnet werden) weitergegeben werden:

a) ☐*(bitte ankreuzen)* **an die Projektleiter* oder eine von diesem beauftragte Stelle zum Zwecke der wissenschaftlichen Auswertung,**

***Anschrift der Leiter des Projektes:** Prof. Dr. med. Beate Herpertz-Dahlmann/PD Dr. med. Jochen Seitz, Klinik für Psychiatrie, Psychosomatik und Psychotherapie des Kindes- und Jugendalters, Uniklinik RWTH Aachen, Pauwelsstraße 30, 52074 Aachen, Tel: 0241/ 80 88737, Fax: 0241/ 80 82544

b) ☐*(bitte ankreuzen)* **im Falle unerwünschter Ereignisse: an den Auftraggeber und die zuständige Ethikkommission.**

2. Außerdem erkläre ich mich damit einverstanden, dass ein autorisierter und zur Verschwiegenheit verpflichteter Beauftragter des Auftraggebersund der Ethikkommission in die beim Studienarzt vorhandenen personenbezogenen Daten

Einsicht nehmen kann, soweit dies für die Überprüfung des Projektes notwendig ist. Für diese Maßnahmen entbinde ich den Studienarzt von der ärztlichen Schweigepflicht.

3. Ich bin einverstanden, dass meine Tochter/mein Sohn für eventuelle spätere Untersuchungen erneut per Brief, Email oder Telefon kontaktiert werden darf. Ich darf selbstverständlich dann erneut frei entscheiden, ob meine Tochter/mein Sohn teilnehmen möchte.

4. Ich bin darüber aufgeklärt worden, dass ich/meine Tochter/mein Sohn jederzeit die Teilnahme an dem Projekt beenden kann. Mir wurde mitgeteilt, dass beim Widerruf der datenschutzrechtlichen Einwilligung die personenbezogenen Daten von mir/meiner Tochter/meines Sohns lediglich anonymisiert werden, da eine Löschung aufgrund gesetzlicher Aufbewahrungspflichten nicht möglich ist. Durch den Widerruf der Einwilligung wird die Rechtmäßigkeit der aufgrund der Einwilligung bis zum Widerruf erfolgten Verarbeitung nicht berührt (Widerruf mit Wirkung für die Zukunft). Der Widerruf ist an den verantwortlichen Studienarzt zu richten.

5. Ich erkläre mich damit einverstanden, dass die Daten von mir/meiner Tochter/meinem Sohn nach Beendigung oder Abbruch des Projektes mindestens zehn Jahre aufbewahrt werden. Danach werden die personenbezogenen Daten gelöscht, soweit nicht gesetzliche, satzungsmäßige oder vertragliche Aufbewahrungsfristen entgegenstehen.

6. Ich bin damit einverstanden, dass Gesundheitsdaten bei mitbehandelnden Ärzten erhoben oder eingesehen werden, soweit dies für die ordnungsgemäße Durchführung und Überwachung des Projektes notwendig ist. Insoweit entbinde ich diese Ärzte von der Schweigepflicht, gemäß § 203 StGB für die v.g. Projektzwecke. (*Falls nicht gewünscht, bitte streichen*.)

7. Ich wünsche ja ☐ / nein ☐ *(bitte ankreuzen)*, dass mein Hausarzt über die Teilnahme von meiner Tochter/meines Sohns an dem o.g. Forschungsprojekt informiert wird.

Name und Anschrift des Hausarztes:

____

____

____

____

Die nachstehenden Angaben müssen vom Erziehungsberechtigten persönlich ausgefüllt werden:

Vollständiger Name der Projektteilnehmerin/Projektteilnehmer in Druckschrift	
Geburtsdatum	

Vollständiger Name der Mutter in Druckschrift	
Ort und Datum	
Unterschrift der Mutter*	

Vollständiger Name des Vaters in Druckschrift	
Ort und Datum	
Unterschrift des Vaters*	

Vollständiger Name des gesetzlichen Vormundes bei Minderjährigen ohne sorgeberechtigte Eltern in Druckschrift	
Ort und Datum	
Unterschrift des gesetzlichen Vormundes bei Minderjährigen ohne sorgeberechtigte Eltern *	

* Durch die Unterschrift unter dieser Teilnehmerinformation und Einwilligungserklärung bestätige ich, dass ich rechtlich dazu ermächtigt bin (das Sorgerecht habe) zu erlauben, dass dieses Kind an diesem Forschungsprojekt teilnimmt. Falls notwendig, werde ich dem Studienarzt Dokumente zum Beweis meines Sorgerechts vorlegen.

Die nachstehenden Angaben müssen vom Studienarzt persönlich ausgefüllt werden:

Ich habe die betroffene Person über Wesen, Bedeutung, Reichweite und Risiken des Forschungsvorhabens aufgeklärt.

Vor- und Nachname des Studienarztes	
Ort und Datum	
Unterschrift des Studienarztes	
