# Supplementary material for: The effects of polyunsaturated fatty acid (PUFA) administration on the microbiome-gut-brain axis in adolescents with anorexia nervosa (the MiGBAN study): study protocol for a longitudinal, double-blind, randomized, placebo-controlled trial
Source: Trials. 2022 Jul 5;23:545. doi: 10.1186/s13063-022-06413-7 (PMC9254435; doi:10.1186/s13063-022-06413-7)
Supplement: Supplementary file 1 — Additional file 1. [file 13063_2022_6413_MOESM1_ESM.zip › migban_consent_form_participantsR1.pdf]

Studienarzt	Studienzentrum	CTC-A-Nr.
Prof. Dr. med. Beate Herpertz-Dahlmann	Klinik für Psychiatrie, Psychotherapie und Psychosomatik des Kindes- und Jugendalters<br>Uniklinik RWTH Aachen	18-118

Information zur Vorbereitung der mündlichen Aufklärung über die Teilnahme an einem Forschungsprojekt

Für Kontrollprobandinnen

Untersuchung der Wirksamkeit der Verabreichung von mehrfach ungesättigten Fettsäuren auf das Mikrobiom bei Jugendlichen mit Anorexia nervosa vs. Placebo

- MIGBAN -

Liebe _______________,

Dieses Informationsblatt soll Dir helfen, vor unserem Gespräch schon etwas über unser Forschungsprojekt zu erfahren. Wir möchten fragen, ob Du bei dem hier beschriebenen Forschungsvorhaben teilnehmen möchtest.

Bei der Anorexia nervosa (Dir vielleicht eher bekannt als Magersucht) handelt es sich um eine Störung, an der ca. 0,5-1 % Prozent aller Mädchen im Alter zwischen 15 und 19 Jahren erkranken. An der Entstehung dieser Störung wirken sowohl Umwelt- als auch genetische Faktoren mit. Die Wiederherstellung und die Aufrechterhaltung eines gesunden Körpers und Gewichtes stellen die wichtigsten Behandlungsziele dar.

Die Anorexia nervosa ist eine schwere Erkrankung, die mit vielen körperlichen und psychischen Begleiterscheinungen einhergeht. So konnten neuere Studien zeigen, dass die Darmflora (auch Mikrobiom genannt), also die Anzahl und Art der Bakterien im Darm, bei Patientinnen und Patienten mit Anorexia nervosa verändert ist und dies mit der Schwere der Erkrankung zusammen zu hängen scheint. Nach wie vor ist aber unklar, inwieweit sich diese Auffälligkeiten durch eine Gewichtszunahme und erfolgreiche Therapie zurückbilden und wie genau der Zusammenhang mit klinischen Auffälligkeiten ist. Um neue Behandlungsmöglichkeiten zu finden, brauchen wir Deine Hilfe.

1 Ziel des Forschungsprojektes

In unserem Projekt möchten wir untersuchen, ob die Einnahme eines sog. Nahrungsergänzungsmittels, nämlich mehrfach ungesättigte Fettsäuren (Omega-3-Fettsäuren) einen Einfluss auf die Darmbakterien bei Jugendlichen mit Anorexia nervosa hat. Des Weiteren wollen wir deinen Stuhl oder Stuhlbestandteile in Tiermodelle transferieren, um dessen Auswirkungen zu erforschen. Die Ergebnisse dieses Forschungsprojektes sollen helfen, die Einflüsse der Ernährung auf die Darmbakterien zu verstehen und langfristig die Behandlung von Patienten und Patientinnen mit Anorexia nervosa zu verbessern.

Wir möchten insgesamt 60 stationäre Patientinnen mit Anorexia nervosa und 30 gesunde Mädchen im Alter zwischen 12 und 20 Jahren für das Projekt untersuchen. Von den 60 Patientinnen erhalten 30 Patientinnen das Nahrungsergänzungsmittel und die anderen 30 Patientinnen ein Placebo. Ein Placebo enthält keinerlei Wirkstoff. Als Kontrolle suchen wir gesunde Kontrollprobanden, die kein Nahrungsergänzungsmittel nehmen sollen.

2 Was sind Omega-3-Fettsäuren?

Omega-3-Fettsäuren gehören zu den mehrfach ungesättigten Fettsäuren und sind für den Körper lebensnotwendig. Der Körper kann Omega-3-Fettsäuren nicht selbst produzieren und muss sie daher über die Nahrung aufnehmen. Sie kommen z. B. in pflanzlichen Lebensmitteln wie Lein, Raps und Walnuss vor und stecken vor allem in Algen und fetten Meeresfischen. Wir verwenden frei verkäufliche, ausschließlich vegane Omega-3 Fettsäuren aus Algen (Opti3 Omega-3 EPA & DHA von Vegetology, Nottingham, UK). Man geht heute davon aus, dass Omega-3-Fettsäuren auch eine positive Wirkung auf das Gehirn haben.

3 Ein- und Ausschlusskriterien

Teilnehmen können gesunde Jugendliche ohne aktuelle psychiatrische Erkrankungen und ohne Essstörungsvergangenheit zwischen 12 und 20 Jahren mit Einverständnis der Eltern oder des gesetzlichen Vormundes bei Minderjährigen ohne sorgeberechtigte Eltern. Nicht teilnehmen können Jugendliche mit organischen Hirnerkrankungen, IQ <80, unzureichenden Deutschkenntnissen, organischen Erkrankungen mit Einfluss auf den Magen-Darmtrakt, wie z.B. Diabetes oder entzündlichen Darmerkrankungen, Schwangerschaft, sowie systemischer Antibiotikaeinnahme in den letzten 6 Wochen.

4 Was wird während der Untersuchung auf Dich zu kommen?

Zunächst überprüft der zuständige Arzt, ob Du die Voraussetzungen zur Teilnahme an dem Projekt erfüllst. Wenn Du und deine Eltern einer Teilnahme zustimmen, wirst Du in das Projekt eingeschlossen.

Anschließend werden zunächst folgende Daten von Dir erhoben/dokumentiert: Alter, Größe, Gewicht, aktuelle Medikation und anderweitige Erkrankungen.

Wir möchten Dich an drei Zeitpunkten des Forschungsprojektes untersuchen. Der erste Untersuchungszeitpunkt findet bei Einschluss in das Projekt statt, der zweite nach 6 Monaten und der letzte 12 Monate nachdem Du mit der Studie begonnen hast.

Als gesunde Kontrollprobandin/gesunder Kontrollproband wird Dir kein Prüfpräparat verabreicht.

Zu jedem Zeitpunkt wird das Gewicht bestimmt, sowie direkt nach Einschluss und beim letzten Zeitpunkt Deine Körpergröße.

Die geplanten Untersuchungen werden in den nachfolgenden Punkten genauer erklärt.

4.1 Fragebögen & neuropsychologische Tests

Bei jedem Untersuchungszeitpunkt bekommst Du kurze Fragebögen zu Deinem Wohlbefinden und Symptomen (Essstörungen, Angst, Depression) ausgehändigt bzw. füllst diese an einem Computer aus (ca. 30 min). Außerdem erfolgen neuropsychologische Tests am Computer (ca. 40 min). Insgesamt würdest du bei jedem Untersuchungszeitpunkt 8 – 9 Fragebögen bei der Teilnahme an dieser Studie ausfüllen. Des Weiteren werden Deine Eltern bei Studienstart und ein Jahr später gebeten, einen Fragebogen auszufüllen.

4.2 Interview zu klinischen Symptomen

Dieses Interview möchten wir bei Aufnahme in die Studie mit Dir führen, es dauert ca. 30 min.

4.3 Stuhlproben

Weiterhin möchten wir Dich bitten, zu jedem Untersuchungszeitpunkt eine Stuhlprobe abzugeben, um die Darmbakterien zu untersuchen. Dabei wird der Stuhl von Dir in einer Plastik-Auffangvorrichtung (Fecotainer) gesammelt, die unter den Toilettensitz eingelegt werden kann und luftdicht verschlossen wird..

4.4 Blutproben

An drei Zeitpunkten benötigen wir Blutproben von Dir. Im Blut werden Entzündungszeichen, Hormone, Botenstoffe, Fettsäuren und Blutzellen untersucht. Eine Untersuchung Deiner Gene findet nicht statt. Es sind keine für Dich individuell relevante, krankheitsbezogene Befunde zu erwarten, die Untersuchungen dienen einzig der Forschung. Die Blut- und Stuhl-Proben werden in Tiefkühltruhen der KJP anonymisiert gelagert und für die Analysen ebenfalls anonym an unsere Kooperationspartner verschickt. Sie werden nach Abschluss der Analysen, spätestens aber nach 10 Jahren vernichtet.

4.5 Ernährungstagebuch

Du wirst gebeten, zwei Tage vor jeder Kontrolle ein Tagebuch über Deine verzehrten Nahrungsmittel zu führen und dieses mitzubringen. Solltest Du innerhalb von 6 Wochen vor diesen Untersuchungen Medikamente oder irgendwann ein Antibiotikum eingenommen haben, möchten wir Dich bitten, auch dies aufzuschreiben.

4.6 Bewegungsmessung (Aktimetrie)

An allen Zeitpunkten wird mittels eines Armbandes ("Aktimeter") für drei Tage die Bewegung gemessen.

4.7 Funktionelles und strukturelles MRT (Magnetresonanztomographie)

Bei den Probandinnen/Probanden ohne Gegenanzeigen wie z.B. eine große Metallzahnspange möchten wir an drei Zeitpunkten eine MRT (Magnetresonanztomografie oder Kernspin)- Untersuchung durchführen.

Bei der MRT-Untersuchung wirst Du auf dem Rücken liegend in den Gerätetunnel, der sowohl am Kopf- als auch am Fußende offen ist, hineingefahren. Der Gerätetunnel besteht aus einem starken Magneten, der in Verbindung mit elektrischen Spulen innere Bilder Deines Körpers erzeugt. Die Untersuchung ist nach heutigem Wissen ungefährlich, lediglich sehr laut, so dass Du Ohrenstopfen bekommst. Eine Strahlenbelastung ist mit dieser Untersuchung nicht verbunden. Die gesamte Untersuchungszeit im MRT beträgt ca. 60 Minuten.

Die Untersuchung beinhaltet anatomische Messungen sowie eine Messung der Gehirnfunktion in Ruhe. Bei allen diesen Messungen sollst Du lediglich entspannt und ruhig daliegen und dich möglichst wenig bewegen. Bei zwei weiteren funktionellen Messungen wirst Du gebeten, wiederholt eine von zwei Figuren auszuwählen. Für die „richtige" Wahl können Punkte gewonnen werden; welche Figur die „Richtige" ist, wechselt nach einem bestimmten Muster, das Du herausfinden sollst. Zwischen den Messungen erfolgt immer ein kurzer Kontakt mit Dir, ob alles in Ordnung ist, und du wirst über die nächste Messung informiert. Während der einzelnen Messungen kannst Du jederzeit mittels eines „Rufballs" mit dem Untersuchungsleiter Kontakt aufnehmen.

Zufallsbefunde: Bei ca. 3% aller jüngeren gesunden Menschen bestehen Abweichungen von der normalen Anatomie, die in der Regel nicht schlimm sind. Um ganz sicher zu gehen, würden wir in einem solchen Fall eine genauere Untersuchung mit Deinen Eltern und Dir besprechen.

5 Welche Risiken gibt es?

Bei Einhaltung aller Sicherheitsvorschriften und Ausschlusskriterien gilt die Magnetresonanz-Tomographie zum heutigen Kenntnisstand als weitgehend ungefährlich. Bei einer Blutentnahme besteht grundsätzlich das Risiko einer Infektion der Einstichstelle, Nervenverletzungen, Hämatome, Schwindel und Unwohlsein.

6 Mögliche Bedeutung der Projektergebnisse und Risiko/Nutzenabwägung

Wie bei allen Experimenten, sind die Ergebnisse zu Beginn eines Forschungsvorhabens nicht absehbar. Die Teilnahme an dem Projekt hat daher voraussichtlich keinen unmittelbaren Nutzen für Dich persönlich. Allerdings könnte unser Projekt für zukünftige Patienten und Patientinnen wichtige Erkenntnisse und eine wesentliche Verbesserung der Behandlung dieser schweren Krankheit erbringen. Mit der Teilnahme an dem Projekt könntest Du vielen zukünftigen Patienten und Patientinnen helfen.

7 Aufwandentschädigung

Für jeden Untersuchungszeitpunkt erhältst Du Bargeld in Höhe von 30 EUR plus Fahrtkosten.

8 Personenbezogene Informationen und Ergebnisse

Die für das Forschungsprojekt wichtigen Daten werden in pseudonymisierter Form gespeichert, ausgewertet und innerhalb der Forschergruppe weitergegeben. Pseudonymisiert bedeutet, dass keine Angaben von Deinem Namen oder Deinen Initialen verwendet werden, sondern nur ein Nummern- und/oder Buchstabencode*.* Die Namensliste, die allein eine Zuordnung der Daten bzw. der Ergebnisse zu deiner Person gestattet, verbleibt unter Verschluss in unserer Klinik. Die Liste wird nach der von der Deutschen Forschungsgemeinschaft geforderten und gesetzlich vorgeschriebenen zehnjährigen Nachweispflicht gelöscht. Bis zu diesem Zeitpunkt wird die Namensliste in einem verschlossenen Raum in unserer Klinik aufbewahrt.
Die im Rahmen des Projektes erhobenen Daten und medizinischen Befunde von Dir werden von einem elektronischen Datensystem erfasst und statistisch ausgewertet. Nach Beendigung des Projektes werden alle Daten nach den derzeit gültigen Richtlinien entsprechend gespeichert und archiviert. Die Verantwortung für die Einhaltung des Datenschutzes im Rahmen des elektronischen Datensystems liegt bei dem Institut für Medizinische Informatik am Universitätsklinikum Aachen, Pauwelsstr. 30, 52074 Aachen. Die Bearbeitung der erhobenen Daten erfolgt in Verantwortung von PD Dr. med. Jochen Seitz, Oberarzt, Klinik für Psychiatrie, Psychotherapie und Psychosomatik des Kindes- und Jugendalters der Uniklinik RWTH Aachen, Neuenhofer Weg 21, 52074 Aachen. Du hast das Recht, Einsicht in Deine Daten zu nehmen, die während der Studie erhoben werden. Solltest Du dabei Fehler in den Daten feststellen, so hast Du das Recht, diese durch den Studienarzt korrigieren zu lassen.
Weiterhin hast Du das Recht auf Auskunft und Überlassung einer Kopie Deiner Daten. Du hast darüber hinaus das Recht, Dich bei einer Aufsichtsbehörde (siehe Punkt 12, Adressen und Kontakte) über den Umgang mit Deinen Daten zu beschweren.
Du triffst Deine Entscheidung nach der mündlichen Aufklärung freiwillig und kannst Dein Einverständnis jederzeit zurücknehmen, ohne dass Dir daraus Nachteile entstehen.

Bei wissenschaftlichen Forschungsprojekten werden persönliche Daten und medizinische Befunde über Dich erhoben. Dabei ist gesetzlich festgelegt, dass diese studienbezogenen

Daten nur ohne Namensnennung gespeichert, weitergegeben und ausgewertet werden dürfen, und zwar:

1.) an die Auftraggeber der Studie zur wissenschaftlichen Auswertung;

Anschrift der Auftraggeber:

Prof. Dr. med. B. Herpertz-Dahlmann/PD Dr. med. Jochen Seitz,
Klinik für Psychiatrie, Psychotherapie und Psychosomatik des Kindes- und Jugendalters,
Uniklinik RWTH Aachen,
Neuenhofer Weg 21, 52074 Aachen
Tel. 0241/8089171

2.) Außerdem kann ein autorisierter und zur Verschwiegenheit verpflichteter Beauftragter des Auftraggebers in die beim Studienarzt vorhandenen personenbezogenen Daten Einsicht nehmen, soweit dies für die Überprüfung der Studie notwendig ist.

9 Versicherung

Die Uniklinik RWTH Aachen und deren an der Studie mitwirkende Mitarbeiter sind haftpflichtversichert für den Fall, dass Du durch deren Verschulden einen Schaden erleidest. Da Du für die zwei Nachuntersuchungen nochmal in die Klinik eingeladen wirst, gibt es zusätzlich für alle Teilnehmer eine Reise-Unfallversicherung für den Weg zur Klinik und zurück mit den Höchstversicherungssummen 100.000 EUR bei Invalidität und 50.000 EUR bei Tod.

10 Finanzierung der Studie

Die Studie wird vom Bundesministerium für Bildung und Forschung finanziert. Sie ist Teil eines europäischen Forschungsprojektes.

11 Habe ich Nachteile, wenn ich nicht an dem Projekt teilnehme?

Nein. Wenn Du nicht an dem Projekt teilnehmen möchtest, entstehen Dir keinerlei Nachteile.

Deine Teilnahme ist zu jeder Zeit freiwillig. Wenn Du nicht mehr teilnehmen möchtest, kannst Du das Deinem Arzt mitteilen.

12 Adressen und Kontakte

Studienzentrum:

Prof. Dr. med. B. Herpertz-Dahlmann/PD Dr. med. Jochen Seitz
Klinik für Psychiatrie, Psychosomatik und Psychotherapie des Kindes- und Jugendalters
Uniklinik RWTH Aachen
Neuenhoferweg 21
52074 Aachen
Tel: 0241 80 89171
Fax: 0241 80 82577

Datenschutzbeauftragter der Studienleitung und Studienzentrum:Joachim Willems
Kullenhofstr. 50
52070 Aachen
Tel: 0241 80 89051
Fax: 0241 80 33 89051

Datenschutzaufsichtsbehörde der Studienleitung und Studienzentrum:

Landesbeauftragte für Datenschutz und Informationsfreiheit (LDI)
Nordrhein-Westfalen
Postfach 20 04 44
40102 Düsseldorf
Tel: 0211/38424-0
Fax: 0211/38424-10

Falls Du weitere Rückfragen hast, kannst Du dich an Dr. med. Jochen Seitz (0241-8089171) wenden oder einen anderen Arzt. Wir wären Dir sehr dankbar, wenn Du Dich zu einer Teilnahme an dem Projekt bereit erklären könntest.

13 Einwilligungserklärung

Probanden Nr.:

Ich habe die Information zu dem Projekt bekommen. Ich weiß jetzt, worum es geht, dass ich jederzeit Fragen stellen kann und meine Teilnahme jederzeit beenden kann. Ich hatte genügend Zeit, mir gemeinsam mit meinen Eltern zu überlegen, ob ich an diesem Projekt teilnehmen möchte. Über die Folgen eines Widerrufs der Gesamtteilnahme oder der datenschutzrechtlichen Einwilligung bin ich aufgeklärt worden.

Ich möchte bei dem Forschungsprojekt mitmachen und willige in die Teilnahme am Forschungsprojekt ein. Eine Kopie der schriftlichen Aufklärung und Einwilligung sowie der Versicherungsbestätigung und –bedingungen der Wege-Unfall-Versicherung habe ich erhalten.

13.1 Datenschutz

Bei wissenschaftlichen Projekten werden persönliche Daten und medizinische Befunde über dich erhoben. Die Weitergabe, Speicherung und Auswertung dieser projektbezogenen Daten erfolgt nach gesetzlichen Bestimmungen und setzt vor Teilnahme an dem Projekt die folgende freiwillige Einwilligung voraus:

1. ☐*(bitte ankreuzen)* **Ich bin damit einverstanden, dass im Rahmen dieses Projektes personenbezogene Daten, insbesondere Angaben über die Gesundheit und ethnische Herkunft, über mich erhoben und in Papierform sowie auf elektronischen Datenträgern in der Klinik für Psychiatrie, Psychosomatik und Psychotherapie des Kindes- und Jugendalters, Uniklinik RWTH Aachen aufgezeichnet werden.**

Soweit erforderlich, dürfen die erhobenen Daten pseudonymisiert (d.h. die Daten können ohne Hinzuziehung zusätzlicher Informationen nicht mehr einer spezifischen betroffenen Person zugeordnet werden) weitergegeben werden:

a) ☐*(bitte ankreuzen)* **an die Projektleiter* oder eine von diesem beauftragte Stelle zum Zwecke der wissenschaftlichen Auswertung,**

***Anschrift der Leiter des Projektes:** Prof. Dr. med. B. Herpertz-Dahlmann/ Dr. med. Jochen Seitz, Klinik für Psychiatrie, Psychosomatik und Psychotherapie des Kindes- und Jugendalters, Uniklinik RWTH Aachen, Pauwelsstraße 30, 52074 Aachen, Tel: 0241/ 80 88737, Fax: 0241/ 80 82544

b) ☐*(bitte ankreuzen)* **im Falle unerwünschter Ereignisse: an den Auftraggeber und die zuständige Ethikkommission.**

2. **Außerdem erkläre ich mich damit einverstanden, dass ein autorisierter und zur Verschwiegenheit verpflichteter Beauftragter des Auftraggebersund der Ethikkommission in die beim Studienarzt vorhandenen personenbezogenen Daten Einsicht nehmen kann, soweit dies für die Überprüfung des Projektes notwendig ist. Für diese Maßnahmen entbinde ich den Studienarzt von der ärztlichen Schweigepflicht.**

3. **Ich bin einverstanden, dass ich für eventuelle spätere Untersuchungen erneut per Brief, Email oder Telefon kontaktiert werden darf. Ich darf selbstverständlich dann erneut frei entscheiden, ob ich teilnehmen möchte.**

4. **Ich bin darüber aufgeklärt worden, dass ich jederzeit die Teilnahme an dem Projekt beenden kann. Mir wurde mitgeteilt, dass beim Widerruf der datenschutzrechtlichen Einwilligung die personenbezogenen Daten von mir lediglich anonymisiert werden, da eine Löschung aufgrund gesetzlicher Aufbewahrungspflichten nicht möglich ist. Durch den Widerruf der Einwilligung wird die Rechtmäßigkeit, der aufgrund der Einwilligung bis zum Widerruf erfolgten Verarbeitung nicht berührt (Widerruf mit Wirkung für die Zukunft). Der Widerruf ist an den verantwortlichen Studienarzt zu richten.**

5. **Ich erkläre mich damit einverstanden, dass die Daten von mir nach Beendigung oder Abbruch des Projektes mindestens zehn Jahre aufbewahrt werden. Danach werden die personenbezogenen Daten gelöscht, soweit nicht gesetzliche, satzungsmäßige oder vertragliche Aufbewahrungsfristen entgegenstehen.**

6. **Ich bin damit einverstanden, dass Gesundheitsdaten bei mitbehandelnden Ärzten erhoben oder eingesehen werden, soweit dies für die ordnungsgemäße Durchführung und Überwachung des Projektes notwendig ist. Insoweit entbinde ich diese Ärzte von der Schweigepflicht, gemäß § 203 StGB für die v.g. Projektzwecke. (*Falls nicht gewünscht, bitte streichen*.)**

7. **Ich wünsche ja ☐ / nein ☐ *(bitte ankreuzen)*, dass mein Hausarzt über die Teilnahme von mir an dem o.g. Forschungsprojekt informiert wird.**

Name und Anschrift des Hausarztes:

____

____

____

____

Die nachstehenden Angaben müssen von Dir persönlich ausgefüllt werden:

Mein Vorname und Nachname	
Geburtsdatum	
Ort und Datum	
Meine Unterschrift*	

Die nachstehenden Angaben müssen vom Studienarzt persönlich ausgefüllt werden:

Ich habe die Eltern/den gesetzlichen Betreuer der betroffenen Person über Wesen, Bedeutung, Reichweite und Risiken des Forschungsvorhabens aufgeklärt.

Vor- und Nachname des Studienarztes	
Ort und Datum	
Unterschrift des Studienarztes	
