# Supplementary material for: The effects of polyunsaturated fatty acid (PUFA) administration on the microbiome-gut-brain axis in adolescents with anorexia nervosa (the MiGBAN study): study protocol for a longitudinal, double-blind, randomized, placebo-controlled trial
Source: Trials. 2022 Jul 5;23:545. doi: 10.1186/s13063-022-06413-7 (PMC9254435; doi:10.1186/s13063-022-06413-7)
Supplement: Supplementary file 1 — Additional file 1. [file 13063_2022_6413_MOESM1_ESM.zip › migban_consent_form_patientsR1.pdf]

**Studienarzt**	**Studienzentrum**	**CTC-A-Nr.**
Prof. Dr. med. Beate Herpertz-Dahlmenn	Klinik für Psychiatrie, Psychotherapie und Psychosomatik des Kindes- und Jugendalters Uniklinik RWTH Aachen	18-118

Information zur Vorbereitung der mündlichen Aufklärung über die Teilnahme an einem Forschungsprojekt

Für Patientinnen

Untersuchung der Wirksamkeit der Verabreichung von mehrfach ungesättigten Fettsäuren auf das Mikrobiom bei Jugendlichen mit Anorexia nervosa vs. Placebo

- MIGBAN -

Liebe _______________,

Dieses Informationsblatt soll Dir helfen, vor unserem Gespräch schon etwas über unser Forschungsprojekt zu erfahren. Wir möchten fragen, ob Du bei dem hier beschriebenen Forschungsvorhaben teilnehmen möchtest.

Bei der Anorexia nervosa (Dir vielleicht eher bekannt als Magersucht) handelt es sich um eine Störung, an der ca. 0,5-1 % Prozent aller Mädchen im Alter zwischen 15 und 19 Jahren erkranken. An der Entstehung dieser Störung wirken sowohl Umwelt- als auch genetische Faktoren mit. Die Wiederherstellung und die Aufrechterhaltung eines gesunden Körpers und Gewichtes stellen die wichtigsten Behandlungsziele dar.

Die Anorexia nervosa ist eine schwere Erkrankung, die mit vielen körperlichen und psychischen Begleiterscheinungen einhergeht. So konnten neuere Studien zeigen, dass die Darmflora (auch Mikrobiom genannt), also die Anzahl und Art der Bakterien im Darm, bei Patientinnen und Patienten mit Anorexia nervosa verändert ist und dies mit der Schwere der Erkrankung zusammen zu hängen scheint. Nach wie vor ist aber unklar, inwieweit sich diese Auffälligkeiten durch eine Gewichtszunahme und erfolgreiche Therapie zurückbilden und wie genau der Zusammenhang mit klinischen Auffälligkeiten ist. Um neue Behandlungsmöglichkeiten zu finden, brauchen wir Deine Hilfe.

**Studienarzt**	**Studienzentrum**	**CTC-A-Nr.**
Prof. Dr. med. Beate Herpertz-Dahlmenn	Klinik für Psychiatrie, Psychotherapie und Psychosomatik des Kindes- und Jugendalters Uniklinik RWTH Aachen	18-118

1 Ziel des Forschungsprojektes

In unserem Projekt möchten wir untersuchen, ob die Einnahme eines sog. Nahrungsergänzungmittels, nämlich mehrfach ungesättigte Fettsäuren (Omega-3-Fettsäuren) einen Einfluss auf die Darmbakterien bei Jugendlichen mit Anorexia nervosa hat. Des Weiteren wollen wir deinen Stuhl oder Stuhlbestandteile in Tiermodelle transferieren, um dessen Auswirkungen zu erforschen. Die Ergebnisse dieses Forschungsprojektes sollen helfen, die Einflüsse der Ernährung auf die Darmbakterien zu verstehen und langfristig die Behandlung von Patienten und Patientinnen mit Anorexia nervosa zu verbessern.

Wir möchten insgesamt 60 stationäre Patientinnen mit Anorexia nervosa und 30 gesunde Mädchen im Alter zwischen 12 und 20 Jahren für das Projekt untersuchen. Von den 60 Patientinnen erhalten 30 Patientinnen das Nahrungsergänzungsmittel und die anderen 30 Patientinnen ein Placebo. Placebo bedeutet ein Scheinmittel, das keinerlei Wirkstoff enthält.

2 Was sind Omega-3-Fettsäuren?

Omega-3-Fettsäuren gehören zu den mehrfach ungesättigten Fettsäuren und sind für den Körper lebensnotwendig. Der Körper kann Omega-3-Fettsäuren nicht selbst produzieren und muss sie daher über die Nahrung aufnehmen. Sie kommen z. B. in pflanzlichen Lebensmitteln wie Lein, Raps und Walnuss vor und stecken vor allem in Algen und fetten Meeresfischen. Wir verwenden frei verkäufliche, ausschließlich vegane Omega-3 Fettsäuren aus Algen (Opti3 Omega-3 EPA & DHA von Vegetology, Nottingham, UK). Man geht heute davon aus, dass Omega-3-Fettsäuren auch eine positive Wirkung auf das Gehirn haben.

3 Ein- und Ausschlusskriterien

Teilnehmen können Patientinnen und Patienten mit typischer oder atypischer Anorexia nervosa (Magersucht), d.h. Patienten/Patientinnen, die alle Merkmale einer Magersucht bis auf das niedrige Gewicht erfüllen, zwischen 12 und 20 Jahren mit Einverständnis der Eltern oder des gesetzlichen Vormundes bei Minderjährigen ohne sorgeberechtigte Eltern. Nicht teilnehmen können Patientinnen und Patienten mit organischen Hirnerkrankungen, Psychosen, bipolaren Störungen, Suchterkrankungen, IQ <80, unzureichenden Deutschkenntnissen, organischen Erkrankungen mit Einfluss auf den Magen-Darmtrakt, wie z.B. Diabetes oder entzündlichen Darmerkrankungen, Schwangerschaft, sowie systemischer Antibiotikaeinnahme in den letzten 6 Wochen.

4 Was wird während der Untersuchung auf Dich zukommen?

Patientinnen werden bei Aufnahme in die Klinik gefragt, ob sie an dem Forschungsprojekt teilnehmen möchten. Wenn Du und Deine Eltern einer Teilnahme zustimmen, entscheidet zunächst ein Computer zufällig, welcher der zwei Behandlungsgruppen Du zugeordnet wirst.

Du erhältst über den Zeitraum von 6 Monaten entweder ein Nahrungsergänzungsmittel mit Omega-3-Fettsäuren oder Placebo, also ein Scheinmedikament in Kapselform. Beide werden 2-mal täglich morgens und abends mit Wasser eingenommen.

Die Einnahme des Nahrungsergänzungsmittels bzw. Placebos erfolgt verblindet. Das bedeutet, weder Du noch Dein Arzt wissen, ob Du das Nahrungsergänzungsmittel oder das Placebo einnimmst.
Das Nahrungsergänzungsmittel und das Placebo enthalten beide Vitamin D3. Vitamin D3 ist wichtig für den Knochenaufbau und würde Dir auch bei einer Nicht-Teilnahme an dem Projekt regelmäßig im Rahmen der normalen Behandlung gegeben werden.

Neben der täglichen Einnahme der Kapseln über 6 Monate möchten wir Dich an vier Zeitpunkten im Rahmen des Forschungsprojektes untersuchen. Der erste Untersuchungszeitpunkt findet nach der stationären Aufnahme bei Einschluss in das Projekt statt, ein weiterer bei stationärer Entlassung und zwei weitere 6 und 12 Monate nachdem Du mit der Studie begonnen hast.

4.1 Fragebögen & neuropsychologische Tests

Bei jedem Untersuchungszeitpunkt bekommst Du kurze Fragebögen zu Deinem Wohlbefinden und Symptomen (Essstörungen, Angst, Depression) ausgehändigt bzw. füllst diese an einem Computer aus (ca. 30min). Außerdem erfolgen neuropsychologische Tests am Computer (ca. 40min). Insgesamt würdest du bei jedem Untersuchungszeitpunkt 8 – 9 Fragebögen bei der Teilnahme an dieser Studie ausfüllen. Des Weiteren werden Deine Eltern bei Aufnahme sowie 6 Monate und ein Jahr nach Deiner Entlassung gebeten, einen Fragebogen auszufüllen.

4.2 Interview zu klinischen Symptomen

Dieses Interview möchten wir bei Aufnahme mit Dir führen, es dauert ca. 45 min.

4.3 Stuhlproben

Weiterhin möchten wir Dich bitten, zu jedem Untersuchungszeitpunkt eine Stuhlprobe abzugeben, um die Darmbakterien zu untersuchen. Dabei wird der Stuhl von Dir in einer Plastik-Auffangvorrichtung (Fecotainer) gesammelt, die unter den Toilettensitz eingelegt werden kann und luftdicht verschlossen wird..

4.4 Blutproben

An vier Zeitpunkten (Aufnahme, Entlassung, 6 und 12 Monate nachdem Du mit der Studie begonnen hast), benötigen wir Blutproben von Dir. Die hierfür notwendigen Blutentnahmen werden möglichst mit den ohnehin stattfindenden Blutentnahmen zur medizinischen Kontrolle zusammengelegt. Im Blut werden Entzündungszeichen, Hormone, Botenstoffe, Fettsäuren und Blutzellen untersucht. Eine Untersuchung Deiner Gene findet nicht statt. Es sind keine für

Dich individuell relevante, krankheitsbezogene Befunde zu erwarten, die Untersuchungen dienen einzig der Forschung. Die Blut- und Stuhl-Proben werden in Tiefkühltruhen der KJP anonymisiert gelagert und für die Analysen ebenfalls anonym an unsere Kooperationspartner verschickt. Sie werden nach Abschluss der Analysen, spätestens aber nach 10 Jahren vernichtet.

4.5 Ernährungstagebuch

Du wirst gebeten, zwei Tage vor der 6- und 12-Monatskontrolle ein Tagebuch über Deine verzehrten Nahrungsmittel zu führen und dieses mitzubringen. Solltest Du innerhalb von 6 Wochen vor diesen Untersuchungen Medikamente oder irgendwann ein Antibiotikum eingenommen haben, möchten wir Dich bitten, auch dies aufzuschreiben.

4.6 Bewegungsmessung (Aktimetrie)

An allen Zeitpunkten wird mittels eines Armbandes ("Aktimeter") für drei Tage die Bewegung gemessen. Diese Information wird nur für die Studie erhoben und geht nicht in die klinische Behandlung ein.

4.7 Magnetresonanztomographie (MRT)

Bei den Patientinnen/Patienten ohne Gegenanzeigen wie z.B. eine große Metallzahnspange (siehe auch separate Fragebögen) möchten wir bei Aufnahme sowie 6 und 12 Monate nach Aufnahme eine MRT (Magnetresonanztomografie oder Kernspin)- Untersuchung des Kopfes im Forschungsscanner der Klinik für Psychiatrie (Siemens, 3 Tesla) im Uniklinikum Aachen durchführen.

Bei der MRT-Untersuchung wirst Du auf dem Rücken liegend in den Gerätetunnel, der sowohl am Kopf- als auch am Fußende offen ist, hineingefahren. Der Gerätetunnel besteht aus einem starken Magneten, der in Verbindung mit elektrischen Spulen innere Bilder Deines Körpers erzeugt. Die Untersuchung ist nach heutigem Wissen ungefährlich, lediglich sehr laut, sodass Du Ohrenstopfen bekommst. Eine Strahlenbelastung ist mit dieser Untersuchung nicht verbunden. Die gesamte Untersuchungszeit im MRT beträgt ca. 60 Minuten.

Die Untersuchung beinhaltet anatomische Messungen sowie eine Messung der Gehirnfunktion in Ruhe. Bei allen diesen Messungen sollst Du lediglich entspannt und ruhig daliegen und dich möglichst wenig bewegen. Bei zwei weiteren funktionellen Messungen wirst Du gebeten, wiederholt eine von zwei Figuren auszuwählen. Für die „richtige“ Wahl können Punkte gewonnen werden; welche Figur die „Richtige“ ist, wechselt nach einem bestimmten Muster, das Du herausfinden sollst. Zwischen den Messungen erfolgt immer ein kurzer Kontakt mit Dir, ob alles in Ordnung ist, und du wirst über die nächste Messung informiert. Während der einzelnen Messungen kannst Du jederzeit mittels eines „Rufballs“ mit dem Untersuchungsleiter Kontakt aufnehmen.

Zufallsbefunde: Bei ca. 3% aller jüngeren gesunden Menschen bestehen Abweichungen von der normalen Anatomie, die in der Regel nicht schlimm sind. Um ganz sicher zu gehen, würden wir in einem solchen Fall eine genauere Untersuchung mit Deinen Eltern und Dir besprechen.

5 Welche Risiken gibt es?

Nach der Einnahme des Nahrungsergänzungsmittels könnte es sein, dass Du aufstoßen musst und einen Fisch-/Algengeschmack im Mund hast. Auch ist es möglich, dass Du ein leichtes Unwohlsein im Bauch verspürst. Solltest Du eine der Nebenwirkungen wahrnehmen, informiere bitte Deinen Arzt, den Du aus der Studie kennst. Bei einer Blutentnahme besteht grundsätzlich das Risiko einer Infektion der Einstichstelle, Nervenverletzungen, Hämatome, Schwindel und Unwohlsein.

Es könnte sein, dass Du der Gruppe zugeordnet wirst, bei denen an drei Zeitpunkten eine MRT-Untersuchung durchgeführt wird. Bei Einhaltung aller Sicherheitsvorschriften und Ausschlusskriterien gilt die Magnetresonanz-Tomographie zum heutigen Kenntnisstand als weitgehend ungefährlich. Solltest Du nach der MRT-Untersuchung irgendwelche Beschwerden haben, melde Dich bei Deinem Arzt.

6 Mögliche Bedeutung der Projektergebnisse und Risiko/Nutzenabwägung

Wie bei allen Experimenten, sind die Ergebnisse zu Beginn eines Forschungsvorhabens nicht absehbar. Die Teilnahme an dem Projekt hat daher voraussichtlich keinen unmittelbaren Nutzen für Dich persönlich. Allerdings könnte unser Projekt für zukünftige Patienten und Patientinnen wichtige Erkenntnisse und eine wesentliche Verbesserung der Behandlung dieser schweren Krankheit erbringen. Mit der Teilnahme an dem Projekt könntest Du vielen zukünftigen Patienten und Patientinnen helfen.

7 Aufwandentschädigung

Für die ersten beiden Untersuchungszeitpunkte erhältst Du jeweils zwei Gutscheine (Einkaufs- oder Kinogutschein) im Wert von 15 EUR (insgesamt 30€), für eine eventuelle Teilnahme im MRT noch einmal einen Gutschein in Höhe von 15 EUR, bei den Nachuntersuchungen 30 EUR plus Fahrtkosten.

8 Personenbezogene Informationen und Ergebnisse

Die für das Forschungsprojekt wichtigen Daten werden in pseudonymisierter Form gespeichert, ausgewertet und innerhalb der Forschergruppe weitergegeben. Pseudonymisiert bedeutet, dass keine Angaben von Deinem Namen oder Deinen Initialen verwendet werden, sondern nur ein Nummern- und/oder Buchstabencode. Die Namensliste, die allein eine Zuordnung der Daten bzw. der Ergebnisse zu deiner Person gestattet, verbleibt unter Verschluss in unserer Klinik. Die Liste wird nach der von der Deutschen

Forschungsgemeinschaft geforderten und gesetzlich vorgeschriebenen zehnjährigen Nachweispflicht gelöscht. Bis zu diesem Zeitpunkt wird die Namensliste in einem verschlossenen Raum in unserer Klinik aufbewahrt. Die Daten werden bei Dir selbst und aus Deinen Krankenakten gesammelt.
Die im Rahmen des Projektes erhobenen Daten und medizinischen Befunde von Dir werden von einem elektronischen Datensystem erfasst und statistisch ausgewertet. Nach Beendigung des Projektes werden alle Daten nach den derzeit gültigen Richtlinien entsprechend gespeichert und archiviert. Die Verantwortung für die Einhaltung des Datenschutzes im Rahmen des elektronischen Datensystems liegt bei dem Institut für Medizinische Informatik am Universitätsklinikum Aachen, Pauwelsstr. 30, 52074 Aachen. Die Bearbeitung der erhobenen Daten erfolgt in Verantwortung von Prof. Dr. med. Beate Herpertz-Dahlmann/PD Dr. med. Jochen Seitz, Klinik für Psychiatrie, Psychotherapie und Psychosomatik des Kindes- und Jugendalters der Uniklinik RWTH Aachen, Neuenhofer Weg 21, 52074 Aachen. Du hast das Recht, Einsicht in Deine Daten zu nehmen, die während der Studie erhoben werden. Solltest Du dabei Fehler in den Daten feststellen, so hast Du das Recht, diese durch den Studienarzt korrigieren zu lassen.
Weiterhin hast Du das Recht auf Auskunft und Überlassung einer Kopie Deiner Daten. Du hast darüber hinaus das Recht, Dich bei einer Aufsichtsbehörde (siehe Punkt 12, Adressen und Kontakte) über den Umgang mit Deinen Daten zu beschweren.
Du triffst Deine Entscheidung nach der mündlichen Aufklärung freiwillig und kannst Dein Einverständnis jederzeit zurücknehmen, ohne dass Dir daraus Nachteile entstehen.

Bei wissenschaftlichen Forschungsprojekten werden persönliche Daten und medizinische Befunde über Dich erhoben. Dabei ist gesetzlich festgelegt, dass diese studienbezogenen Daten nur ohne Namensnennung gespeichert, weitergegeben und ausgewertet werden dürfen, und zwar:

1.) an die Auftraggeber der Studie zur wissenschaftlichen Auswertung;

Anschrift der Auftraggeber:
Prof. Dr. med. Beate Herpertz-Dahlmann/PD Dr. med. Jochen Seitz,
Klinik für Psychiatrie, Psychotherapie und Psychosomatik des Kindes- und Jugendalters,
Uniklinik RWTH Aachen,
Neuenhofer Weg 21, 52074 Aachen
Tel. 0241/8089171

2.) Außerdem kann ein autorisierter und zur Verschwiegenheit verpflichteter Beauftragter des Auftraggebers in die beim Studienarzt vorhandenen personenbezogenen Daten Einsicht nehmen, soweit dies für die Überprüfung der Studie notwendig ist.

9 Versicherung

Die Uniklinik RWTH Aachen und deren an der Studie mitwirkende Mitarbeiter sind haftpflichtversichert für den Fall, dass Du durch deren Verschulden einen Schaden erleidest.

Da Du für die zwei Nachuntersuchungen nochmal in die Klinik eingeladen wirst, gibt es zusätzlich für alle Teilnehmer eine Reise-Unfallversicherung für den Weg zur Klinik und zurück mit den Höchstversicherungssummen 100.000 EUR bei Invalidität und 50.000 EUR bei Tod.

10 Finanzierung der Studie

Die Studie wird vom Bundesministerium für Bildung und Forschung finanziert. Sie ist Teil eines europäischen Forschungsprojektes.

11 Habe ich Nachteile, wenn ich nicht an dem Projekt teilnehme?

Nein. Wenn Du nicht an dem Projekt teilnehmen möchtest, entstehen Dir keinerlei Nachteile. Du wirst dann gemäß den Anordnungen Deines Arztes und der regulären medizinischen Versorgung behandelt.

Deine Teilnahme ist zu jeder Zeit freiwillig. Wenn Du nicht mehr teilnehmen möchtest, kannst Du das Deinem Arzt mitteilen. Ein Abbruch der Projektteilnahme führt zu keiner nachteiligen Behandlung, Du wirst gemäß der Standardversorgung weiterbehandelt.

12 Adressen und Kontakte

Studienzentrum:

Prof. Dr. med. Beate Herpertz-Dahlmann/PD Dr. med. Jochen Seitz
Klinik für Psychiatrie, Psychosomatik und Psychotherapie des Kindes- und Jugendalters
Uniklinik RWTH Aachen
Neuenhoferweg 21
52074 Aachen
Tel: 0241 80 89171
Fax: 0241 80 82577

Datenschutzbeauftragter der Studienleitung und Studienzentrum:

Joachim Willems
Kullenhofstr. 50
52070 Aachen
Tel: 0241 80 89051
Fax: 0241 80 33 89051

Datenschutzaufsichtsbehörde der Studienleitung und Studienzentrum:

Landesbeauftragte für Datenschutz und Informationsfreiheit (LDI)
Nordrhein-Westfalen
Postfach 20 04 44
40102 Düsseldorf

**Studienarzt**	**Studienzentrum**	**CTC-A-Nr.**
Prof. Dr. med. Beate Herpertz-Dahlmenn	Klinik für Psychiatrie, Psychotherapie und Psychosomatik des Kindes- und Jugendalters Uniklinik RWTH Aachen	18-118

Tel: 0211/38424-0
Fax: 0211/38424-10

Falls Du weitere Rückfragen hast, kannst Du dich an PD Dr. med. Jochen Seitz (0241-8089171) wenden oder einen anderen Arzt. Wir wären Dir sehr dankbar, wenn Du Dich zu einer Teilnahme an dem Projekt bereit erklären könntest. Damit hilfst Du wahrscheinlich vielen, die später an Magersucht erkranken.

**Studienarzt**	**Studienzentrum**	**CTC-A-Nr.**
Prof. Dr. med. Beate Herpertz-Dahlmenn	Klinik für Psychiatrie, Psychotherapie und Psychosomatik des Kindes- und Jugendalters Uniklinik RWTH Aachen	18-118

13 Einwilligungserklärung

Patienten Nr.:

Ich habe die Information zu dem Projekt bekommen. Ich weiß jetzt, worum es geht, dass ich jederzeit Fragen stellen kann und meine Teilnahme jederzeit beenden kann. Ich hatte genügend Zeit, mir gemeinsam mit meinen Eltern zu überlegen, ob ich an diesem Projekt teilnehmen möchte. Über die Folgen eines Widerrufs der Gesamtteilnahme oder der datenschutzrechtlichen Einwilligung bin ich aufgeklärt worden.

Ich möchte bei dem Forschungsprojekt mitmachen und willige in die Teilnahme am Forschungsprojekt ein. Eine Kopie der schriftlichen Aufklärung und Einwilligung sowie der Versicherungsbestätigung und –bedingungen der Wege-Unfall-Versicherung habe ich erhalten.

13.1 Datenschutz

Bei wissenschaftlichen Projekten werden persönliche Daten und medizinische Befunde über Dich erhoben. Die Weitergabe, Speicherung und Auswertung dieser projektbezogenen Daten erfolgt nach gesetzlichen Bestimmungen und setzt vor Teilnahme an dem Projekt die folgende freiwillige Einwilligung voraus:

1. ☐*(bitte ankreuzen)* **Ich erkläre mich damit einverstanden, dass im Rahmen dieses Projektes personenbezogene Daten, insbesondere Angaben über die Gesundheit und ethnische Herkunft, über mich erhoben und in Papierform sowie auf elektronischen Datenträgern in der Klinik für Psychiatrie, Psychosomatik und Psychotherapie des Kindes- und Jugendalters, Uniklinik RWTH Aachen aufgezeichnet werden.**

Soweit erforderlich, dürfen die erhobenen Daten pseudonymisiert (d.h. die Daten können ohne Hinzuziehung zusätzlicher Informationen nicht mehr einer spezifischen betroffenen Person zugeordnet werden) weitergegeben werden:

a) ☐*(bitte ankreuzen)* **an die Projektleiter* oder eine von diesem beauftragte Stelle zum Zwecke der wissenschaftlichen Auswertung,**

***Anschrift der Leiter des Projektes:** Prof. Dr. med. Beate Herpertz-Dahlmann/ Dr. med. Jochen Seitz, Klinik für Psychiatrie, Psychosomatik und Psychotherapie des Kindes- und Jugendalters, Uniklinik RWTH Aachen, Pauwelsstraße 30, 52074 Aachen, Tel: 0241/ 80 88737, Fax: 0241/ 80 82544

b) ☐*(bitte ankreuzen)* **im Falle unerwünschter Ereignisse: an den Auftraggeber und die zuständige Ethikkommission.**

2. Außerdem erkläre ich mich damit einverstanden, dass ein autorisierter und zur Verschwiegenheit verpflichteter Beauftragter des Auftraggebers und der Ethikkommission in die beim Studienarzt vorhandenen personenbezogenen Daten Einsicht nehmen kann, soweit dies für die Überprüfung des Projektes notwendig ist. Für diese Maßnahmen entbinde ich den Studienarzt von der ärztlichen Schweigepflicht.

3. Ich bin einverstanden, dass ich für eventuelle spätere Untersuchungen erneut per Brief, Email oder Telefon kontaktiert werden darf. Ich darf selbstverständlich dann erneut frei entscheiden, ob ich teilnehmen möchte.

4. Ich bin darüber aufgeklärt worden, dass ich jederzeit die Teilnahme an dem Projekt beenden kann. Mir wurde mitgeteilt, dass beim Widerruf der datenschutzrechtlichen Einwilligung die personenbezogenen Daten von mir lediglich anonymisiert werden, da eine Löschung aufgrund gesetzlicher Aufbewahrungspflichten nicht möglich ist. Durch den Widerruf der Einwilligung wird die Rechtmäßigkeit, der aufgrund der Einwilligung bis zum Widerruf erfolgten Verarbeitung nicht berührt (Widerruf mit Wirkung für die Zukunft). Der Widerruf ist an den verantwortlichen Studienarzt zu richten.

5. Ich erkläre mich damit einverstanden, dass die Daten von mir nach Beendigung oder Abbruch des Projektes mindestens zehn Jahre aufbewahrt werden. Danach werden die personenbezogenen Daten gelöscht, soweit nicht gesetzliche, satzungsmäßige oder vertragliche Aufbewahrungsfristen entgegenstehen.

6. Ich bin damit einverstanden, dass Gesundheitsdaten bei mitbehandelnden Ärzten erhoben oder eingesehen werden, soweit dies für die ordnungsgemäße Durchführung und Überwachung des Projektes notwendig ist. Insoweit entbinde ich/wir diese Ärzte von der Schweigepflicht, gemäß § 203 StGB für die v.g. Projektzwecke. (*Falls nicht gewünscht, bitte streichen*.)

7. Ich wünsche ja ☐ / nein ☐ *(bitte ankreuzen)*, dass mein Hausarzt über die Teilnahme von mir an dem o.g. Forschungsprojekt informiert wird.

Name und Anschrift des Hausarztes:

____

____

____

Die nachstehenden Angaben müssen von Dir persönlich ausgefüllt werden:

Mein Vorname und Nachname	
Geburtsdatum	
Ort und Datum	
Meine Unterschrift*	

Die nachstehenden Angaben müssen vom Studienarzt persönlich ausgefüllt werden:

Mit meiner Unterschrift bestätige ich, dass ich dieser Patientin Natur, Ziel und mögliche Komplikationen dieses Projektes erklärt habe. Nach körperlicher und psychischer Verfassung waren die Patientin in der Lage, Wesen, Bedeutung und Tragweite des Projektes einzusehen und Ihren Willen hiernach zu bestimmen.

Vor- und Nachname des Studienarztes	
Ort und Datum	
Unterschrift des Studienarztes	
